# Liposomal UHRF1 siRNA shows lung fibrosis treatment potential through regulation of fibroblast activation

**DOI:** 10.1172/jci.insight.162831

**Published:** 2022-11-22

**Authors:** Demin Cheng, Yue Wang, Ziwei Li, Haojie Xiong, Wenqing Sun, Sichuan Xi, Siyun Zhou, Yi Liu, Chunhui Ni

**Affiliations:** 1 Department of Occupational Medical and Environmental Health, Key Laboratory of Modern Toxicology, Ministry of Education, Center for Global Health, School of Public Health, Nanjing Medical University, Nanjing, China.; 2 Thoracic Epigenetics Section, Thoracic Surgery Branch, National Cancer Institute, NIH, Bethesda, Maryland, USA.; 3 Gusu School, Nanjing Medical University, Nanjing, China.

**Keywords:** Pulmonology, Fibrosis

## Abstract

Pulmonary fibrosis is a chronic and progressive interstitial lung disease associated with the decay of pulmonary function, which leads to a fatal outcome. As an essential epigenetic regulator of DNA methylation, the involvement of ubiquitin-like containing PHD and RING finger domains 1 (UHRF1) in fibroblast activation remains largely undefined in pulmonary fibrosis. In the present study, we found that TGF-β1–mediated upregulation of UHRF1 repressed beclin 1 via methylated induction of its promoter, which finally resulted in fibroblast activation and lung fibrosis both in vitro and in vivo. Moreover, knockdown of UHRF1 significantly arrested fibroblast proliferation and reactivated beclin 1 in lung fibroblasts. Thus, intravenous administration of UHRF1 siRNA–loaded liposomes significantly protected mice against experimental pulmonary fibrosis. Accordingly, our data suggest that UHRF1 might be a novel potential therapeutic target in the pathogenesis of pulmonary fibrosis.

## Graphical abstract

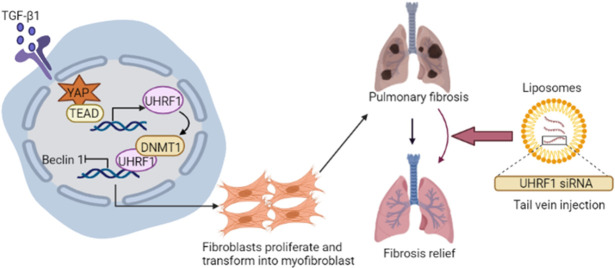

## Introduction

Pulmonary fibrosis is a chronic, progressive, and incurable end-stage lung interstitial disease associated with poor lung function, and this disease remains a considerable burden to public health (1). As a disease of multiple interacting genetic and environmental risk factors, pulmonary fibrosis is characterized by excessive fibroblast proliferation and extracellular matrix (ECM) protein accumulation (2). Despite some advances over the past few decades, the underlying molecular mechanisms of pulmonary fibrosis are not clear, and effective intervention therapies are limited. Consequently, there is an urgent need for establishment of a novel, effective, and available treatment for pulmonary fibrosis.

Activated fibroblasts proliferate and migrate widely to occupy injured areas, which have been recognized as the major source of ECM deposition (3). The persistent proliferation and differentiation of fibroblasts into contractile mesenchymal cells, observed as a high level of α-SMA (a myofibroblast marker), termed fibroblast-to-myofibroblast transition, is essential for pulmonary fibrosis (4, 5). During lung injury, fibroblasts are exposed to various profibrotic cytokines, including TGF-β1, PDGF, and TNF-α. Notably, TGF-β1–induced fibroblast-to-myofibroblast transition is the principal mechanism in the fibrogenic process. Thus, optimal efforts are currently devoted to identifying therapies that can block this biological pathway for the treatment of pulmonary fibrosis.

Ubiquitin-like containing PHD and RING finger domains 1 (UHRF1), also known as NP95 in mice and ICB90 in humans, exerts a significant role in regulating and maintaining DNA methylation by recruiting DNA methyltransferase 1 (Dnmt1) to hemimethylated DNA sites (6). Besides binding to enzymes for DNA methylation, UHRF1 can also mediate the deacetylation of histones (such as H3K9) (7). Recent studies have reported that UHRF1 is dysregulated in many tumors, including non–small cell lung cancer, bladder cancer, and prostate cancer (8, 9). However, the function and expression of UHRF1 in fibroblasts have not been discussed in pulmonary fibrosis.

In recent years, gene therapeutic agents (such as siRNA) have made rapid progress in treating various diseases. For example, liposomes are bilayer or multilayer lipid vesicles that introduce lipid-soluble and water-soluble drugs and usually act as established drug carriers (10, 11). Moreover, cationic liposomes have become one of the most visible siRNA delivery vehicles, with few side effects, including toxicity, biodegradability, and inflammatory response (12). For pulmonary fibrosis, it has been reported that liposomes might prefer to accumulate in the fibrotic lesion after administration (13). Therefore, we attempted to suppress silica- or bleomycin-induced (BLM-induced) mouse pulmonary fibrosis with UHRF1 siRNA–loaded cationic liposomes.

In this study, we found that TGF-β1–mediated upregulation of UHRF1 repressed beclin 1 via methylated induction of its promoter, which resulted in fibroblast activation and lung fibrosis both in vitro and in vivo. Moreover, depletion of UHRF1 significantly arrested fibroblast proliferation and reactivated beclin 1 in lung fibroblasts. We found that intravenous administration of UHRF1 siRNA–loaded liposomes significantly protected mice against experimental pulmonary fibrosis. Accordingly, our data suggested that UHRF1 might be a novel, potential therapeutic target in the pathogenesis of pulmonary fibrosis.

## Results

### Upregulation of UHRF1 in TGF-β1–stimulated fibroblasts and fibrotic lungs.

**Figure 1:**
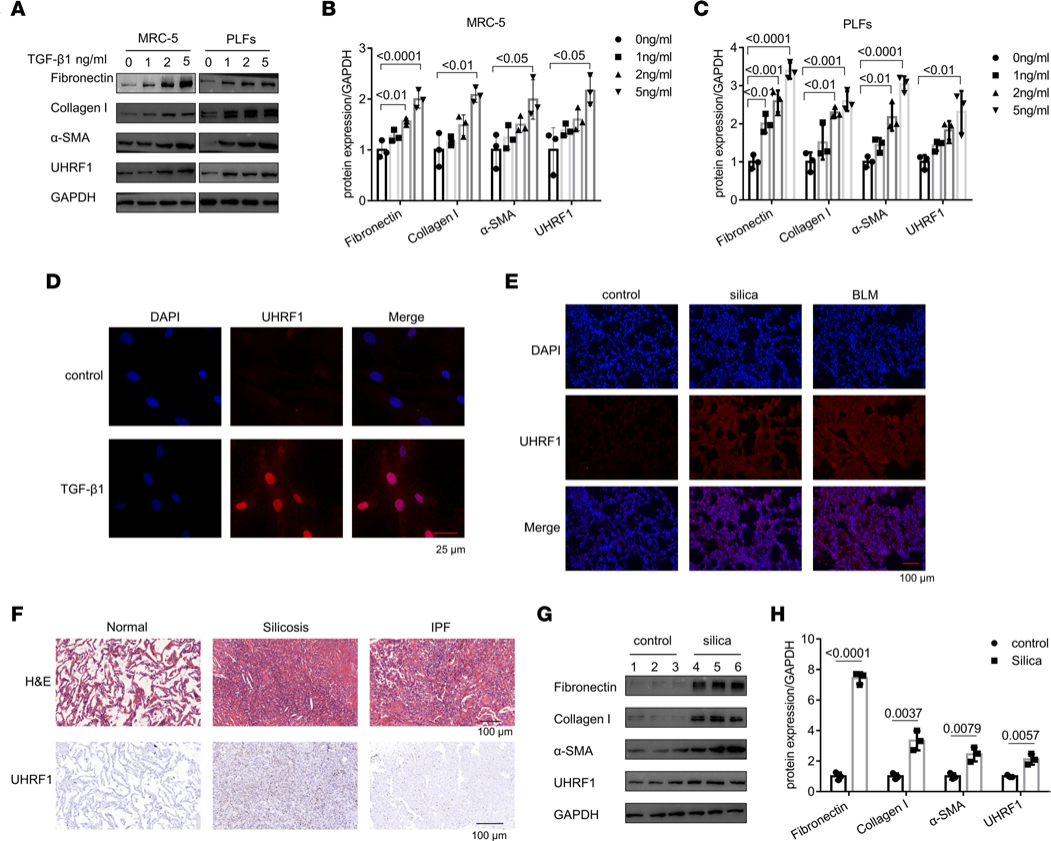
UHRF1 is overexpressed in TGF-β1–stimulated fibroblasts and fibrotic lungs. (**A**–**C**) Western blot and corresponding densitometry analysis of fibronectin, collagen I, α-SMA, and UHRF1 in TGF-β1–treated (0, 1, 2, 5 ng/mL for 48 hours) MRC-5 cells and PLFs. Data are shown as the mean ± SEM (*n* = 3 in each group). (**D**) Immunofluorescence staining of UHRF1 in MRC-5 cells. Red represents UHRF1; blue represents nuclear DNA staining by DAPI. (**E**) Immunofluorescence staining of UHRF1 in mouse lung tissues. Red represents UHRF1; blue represents DAPI. (**F**) Representative results of H&E and UHRF1 IHC staining in lung sections from patients with silicosis and idiopathic pulmonary fibrosis (IPF) and normal participants. (**G** and **H**) Western blot and densitometric analysis of fibronectin, collagen I, α-SMA, and UHRF1 protein in saline- or silica-treated mouse lung tissues. Data are shown as the mean ± SEM (*n* = 3 in each group). Scale bar: 25 μm (**D**); 100 μm (**E** and **F**). *P* values were from (**B** and **C**) a 1-way ANOVA post hoc test with Tukey’s correction or (**H**) 2-tailed unpaired Student’s *t* test.

First, we examined UHRF1 expression in fibroblasts induced with different concentrations of TGF-β1 (0, 1, 2, and 5 ng/mL) for 48 hours. As expected, both fibrotic markers (fibronectin, collagen I, and α-SMA) and UHRF1 were increased in a dose-response manner (Figure 1, A–C) in human embryonic lung fibroblasts (MRC-5 cells) and primary murine lung fibroblasts (PLFs). In addition, UHRF1 mRNA levels were markedly upregulated in fibrotic mouse lung tissues and TGF-β1–treated fibroblasts (Supplemental Figure 1, A and B; supplemental material available online with this article; https://doi.org/10.1172/jci.insight.162831DS1). Immunofluorescence assays revealed that UHRF1 was highly expressed following TGF-β1 treatment in fibroblasts and silica- and BLM-induced fibrotic lung tissues, respectively (Figure 1, D and E). Moreover, the coimmunostaining results further reinforced the conclusion that UHRF1 was highly expressed in α-SMA^+^ myofibroblasts in silica-induced mouse lung tissues (Supplemental Figure 1C). These data supported that UHRF1 was activated in fibroblasts. Additionally, H&E and IHC staining for UHRF1 reinforced that UHRF1 was highly expressed in patients with silicosis, mainly in the fibrotic area (Figure 1F). Furthermore, overexpression of UHRF1 was noted in the mouse tissues following silica and BLM induction, along with increased expression of fibrotic markers (Figure 1, G and H, and Supplemental Figure 1, D and E). Collectively, these findings suggested that UHRF1 may play an essential role in regulating the procession of pulmonary fibrosis.

### UHRF1 played an essential role in TGF-β1–induced lung fibroblast activation.

**Figure 2:**
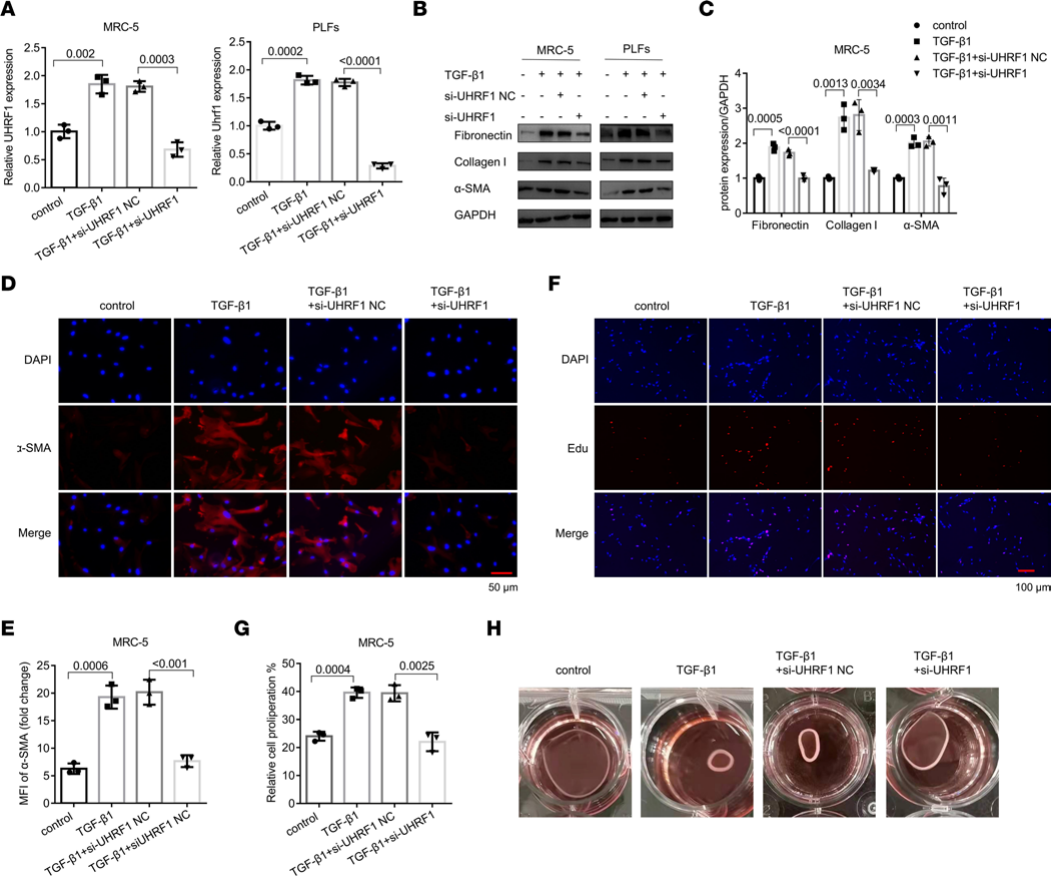
UHRF1 regulates TGF-β1–induced lung fibroblast proliferation. (**A**) qRT-PCR analysis of UHRF1 expression in MRC-5 cells and PLFs transfected with UHRF1 siRNA or its negative control (NC) siRNA, Data are shown as the mean ± SEM (*n* = 3 in each group). (**B** and **C**) Western blot and corresponding densitometry analysis of fibronectin, collagen I, and α-SMA in MRC-5 cells and PLFs transfected with UHRF1 siRNA and its negative control siRNA and then treated with 5 ng/mL TGF-β1 for 48 hours. Data are shown as the mean ± SEM (*n* = 3 in each group). (**D**) The expression of α-SMA was detected by immunofluorescence staining in MRC-5 cells transfected with UHRF1 siRNA and its negative control siRNA and then treated with 5 ng/mL TGF-β1 for 48 hours. (**E**) Mean fluorescence intensity of α-SMA in MRC-5 cells from the different groups. Data are shown as the mean ± SEM (*n* = 3 in each group). (**F** and **G**) Proliferation of MRC-5 cells transfected with UHRF1 siRNA and its negative control siRNA, as assessed by EdU assays. Data are shown as the mean ± SEM (*n* = 3 in each group). (**H**) Effect of UHRF1 siRNA and its negative control siRNA on the contractility of TGF-β1–induced fibroblasts. Scale bar: 50 μm (**D**); 100 μm (**F**). (**A**, **C**, **E**, and **G**) *P* values were from a 1-way ANOVA post hoc test with Tukey’s correction.

Next, to investigate whether UHRF1 is required for fibroblast activation, we performed loss-of-function experiments using siRNAs. First, the mRNA and protein expression levels of UHRF1 were significantly downregulated in fibroblasts by UHRF1 siRNA (Figure 2A and Supplemental Figure 2A). Then, we observed decreases in fibronectin, collagen I, and α-SMA protein levels after UHRF1 knockdown (Figure 2, B and C, and Supplemental Figure 2B). As a selective UHRF1 inhibitor, compound NSC232003 exerted markedly antifibrotic effects by inhibiting the protein expression of fibrotic markers (Supplemental Figure 2C). Moreover, UHRF1 knockdown substantially reduced the expression of α-SMA via α-SMA staining in lung fibroblasts (Figure 2, D and E, and Supplemental Figure 2D). The EdU and CCK8 assays revealed that knockdown of UHRF1 inhibited lung fibroblast proliferation activity stimulated by TGF-β1 (Figure 2, F and G, and Supplemental Figure 2, E–G). The collagen gel contraction assay further confirmed that UHRF1 knockdown inhibited TGF-β1–induced fibroblast-mediated collagen contraction (Figure 2H). These results indicated that the low expression of UHRF1 inhibited fibroblast activation.

### The YAP/TEAD pathway contributed to the elevated expression of UHRF1 in activated fibroblasts.

**Figure 3:**
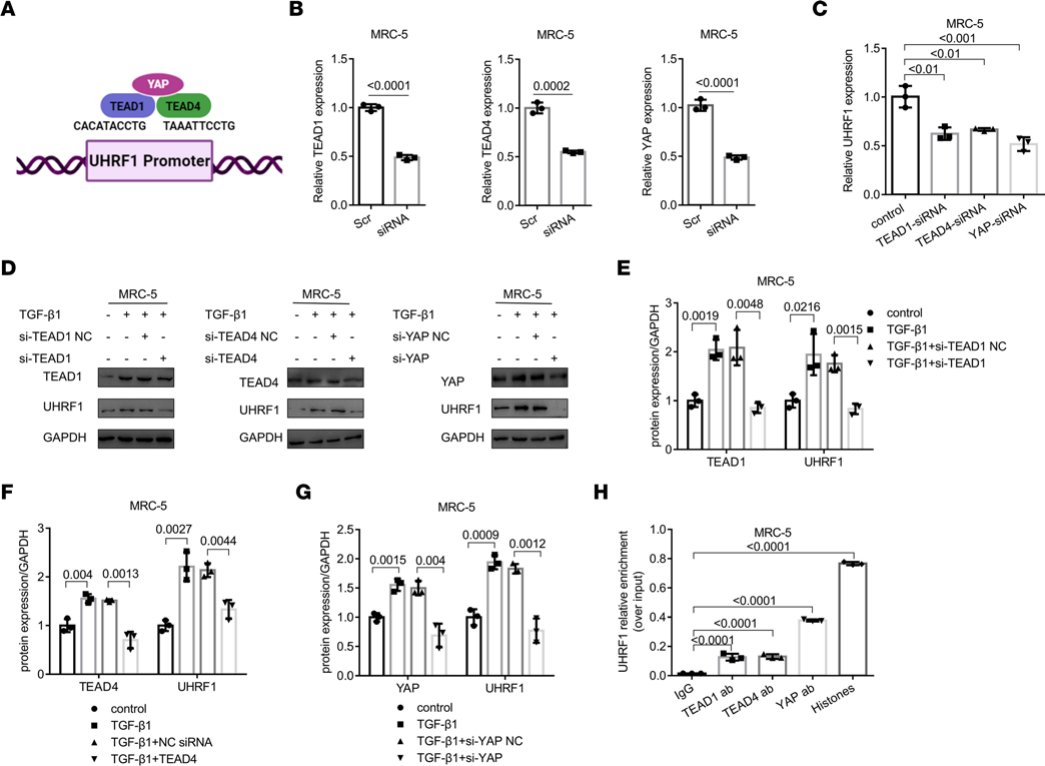
The YAP/TEAD pathway contributes to the expression of UHRF1 in fibroblast activation. (**A**) The potential binding sites (including TEAD1 and TEAD4) at the UHRF1 promoter region by using the JASPAR database. (**B**) Target siRNA transfection significantly decreased the expression of TEAD1, TEAD4, and YAP in MRC-5 cells. Data are shown as the mean ± SEM (*n* = 3 in each group). (**C**) qRT-PCR detection of UHRF1 expression in MRC-5 cells after transfection with TEAD1, TEAD4, and YAP siRNA. Data are shown as the mean ± SEM (*n* = 3 in each group). (**D**–**G**) Western blot and corresponding densitometry analysis of TEAD1, TEAD4, YAP, and UHRF1 in MRC-5 cells and PLFs transfected with TEAD1, TEAD4, and YAP siRNA and their negative control (NC) siRNA and then treated with 5 ng/mL TGF-β1 for 48 hours. Data are shown as the mean ± SEM (*n* = 3 in each group). (**H**) Chromatin was harvested for immunoprecipitation with IgG, an anti-TEAD1 antibody, an anti-TEAD4 antibody, an anti-YAP antibody, and an anti–histone H3 antibody. The expression of UHRF1 was detected by qRT-PCR analysis. Data are shown as the mean ± SEM (*n* = 3 in each group). *P* values were from (**B**) a 2-tailed unpaired Student’s *t* test and (**C** and **E**–**H**) a 1-way ANOVA post hoc test with Tukey’s correction.

We have confirmed the antifibrotic role of UHRF1 on TGF-β1–induced fibroblasts, but the mechanism behind this regulatory effect remains unclear. By using the JASPAR database (https://jaspar.genereg.net/), we predicted that TEAD1 and TEAD4, the members of the TEAD protein family, could directly bind to the UHRF1 promoter region (Figure 3A). Previous studies have indicated that the YAP/TEAD pathway involved fibroblast activation and promoted pulmonary fibrosis (14). Therefore, we hypothesized that the YAP/TEAD pathway might contribute to the upregulation of UHRF1 in the process of fibroblast activation. First, RNA interference was used to knockdown the mRNA levels of TEAD1, TEAD4, and YAP in lung fibroblasts (Figure 3B and Supplemental Figure 3A). Interference with TEAD1, TEAD4, and YAP expression resulted in marked downregulation of UHRF1 mRNA and protein levels (Figure 3, C–G, and Supplemental Figure 3, B–F). Furthermore, ChIP experiments indicated that UHRF1 is the key downstream target of the YAP/TEAD pathway (Figure 3H). Altogether, these data suggested that UHRF1 mediated the activation of fibroblasts through the YAP/TEAD pathway.

### UHRF1 induced de novo promoter-specific methylation to suppress beclin 1 in lung fibroblasts.

**Figure 4:**
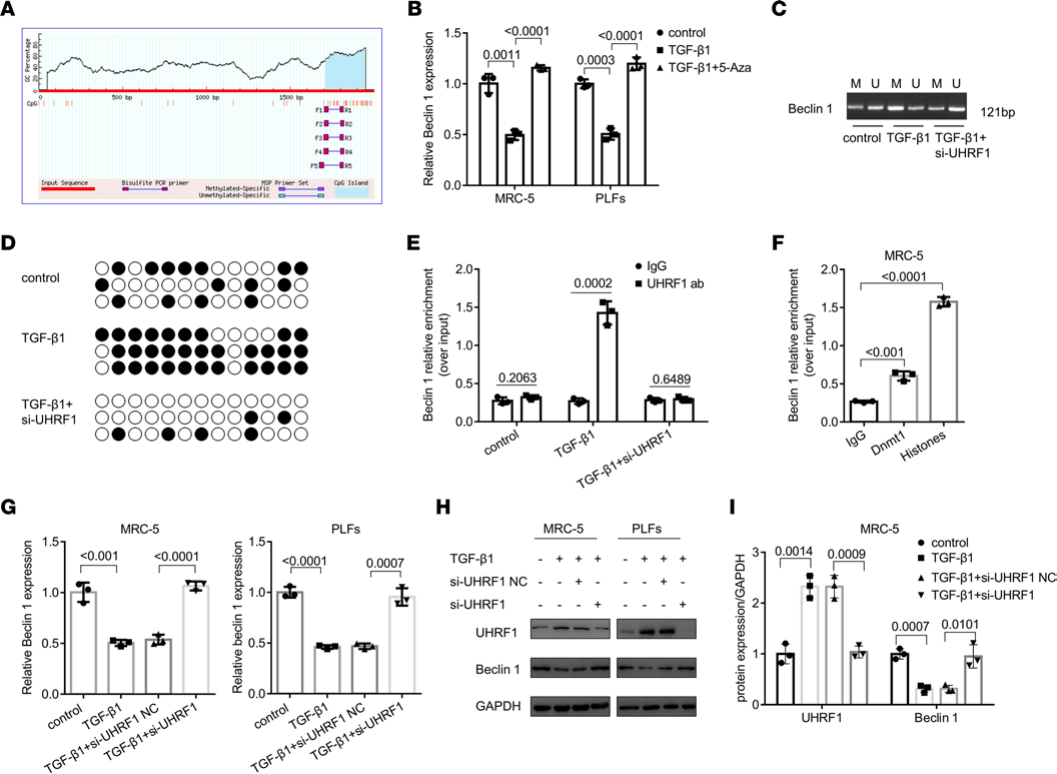
UHRF1 epigenetically mediates beclin 1 methylation in lung fibroblasts. (**A**) Prediction of beclin 1 methylation CpG sites by using http://www.urogene.org/methprimer/ (**B**) Beclin 1 expression in MRC-5 cells and PLFs before and after 5-aza-2′-deoxycytidine treatment. Data are shown as the mean ± SEM (*n* = 3 in each group). (**C**) CpG island methylation status of the beclin 1 gene analyzed by a methylation-specific PCR assay in UHRF1 siRNA–treated PLFs. (**D**) A bisulfite sequencing assay was performed to reveal the CpG methylation status in the beclin 1 promoter region in the PLFs. (**E**) Chromatin was harvested for immunoprecipitation with IgG, an anti-UHRF1 antibody, after being transfected with TGF-β1 or TGF-β1 plus UHRF1 siRNA. The expression of beclin 1 was detected by qRT-PCR analysis. Data are shown as the mean ± SEM (*n* = 3 in each group). (**F**) Chromatin was harvested for immunoprecipitation with IgG, an anti-Dnmt1 antibody, and an anti–histone H3 antibody after TGF-β1 treatment in the fibroblasts. The expression of beclin 1 was detected by qRT-PCR analysis. Data are shown as the mean ± SEM (*n* = 3 in each group). (**G**) qRT-PCR analysis of beclin 1 expression in MRC-5 cells and PLFs after transfection with UHRF1 siRNA or its negative control (NC) siRNA. Data are shown as the mean ± SEM (*n* = 3 in each group). (**H** and **I**) Western blot and corresponding densitometry analysis of UHRF1 and beclin 1 in MRC-5 cells and PLFs transfected with UHRF1 siRNA and its negative control (NC) siRNA and then treated with 5 ng/mL TGF-β1 for 48 hours. Data are shown as the mean ± SEM (*n* = 3 in each group). *P* values were from (**E**) a 2-tailed unpaired Student’s *t* test and (**B**, **F**, **G**, and **I**) a 1-way ANOVA post hoc test with Tukey’s correction.

UHRF1 is acknowledged as a key regulator of DNA methylation combined with Dnmt1 (15), and a CpG island also exists on the beclin 1 promoter (Figure 4A). Thus, we asked whether UHRF1-mediated beclin 1 expression is associated with DNA methylation. Using 5-aza-dC, an inhibitor of methylation, we observed that beclin 1 expression was augmented compared with that in the TGF-β1 treatment group (Figure 4B). Next, we conducted a methylation-specific PCR assay and revealed that low expression of UHRF1 contributed to a remarkable decrease of beclin 1 promoter methylation (Figure 4C). Bisulfite-sequencing PCR was performed to detect beclin 1 CpG island methylation status, and the results illustrated that the knockdown of UHRF1 decreased the methylation of CpG nucleotides with elevated expression of beclin 1 (Figure 4D). Furthermore, ChIP-qPCR assays for beclin 1 were performed with chromatin from lung fibroblasts treated with anti-UHRF1 antibody in UHRF1-knockdown and TGF-β1–treated cells. As it turned out, UHRF1 could directly bind to the beclin 1 promoter in the TGF-β1–treated group compared with that in the UHRF1 siRNA–treated group (Figure 4E). Similarly, the interaction of beclin 1 and Dnmt1 also was identified by ChIP assay (Figure 4F). Thus, we concluded that UHRF1 regulated beclin 1 expression by recruiting Dnmt1 to its promoter to mediate beclin 1 methylation. Besides, UHRF1 knockdown significantly reversed TGF-β1–induced beclin 1 downregulation both at mRNA and protein levels (Figure 4, G–I, and Supplemental Figure 4A). compound NSC232003 could also increase the beclin 1 protein level (Supplemental Figure 4, B–D). Meanwhile, immunofluorescence staining for beclin 1 showed that UHRF1 knockdown reversed the expression of beclin 1 in lung fibroblasts (Supplemental Figure 4, E and F). These results prove that UHRF1 raised beclin 1 expression by epigenetically mediating its methylation.

### Beclin 1 downregulation arrested fibroblast proliferation.

**Figure 5:**
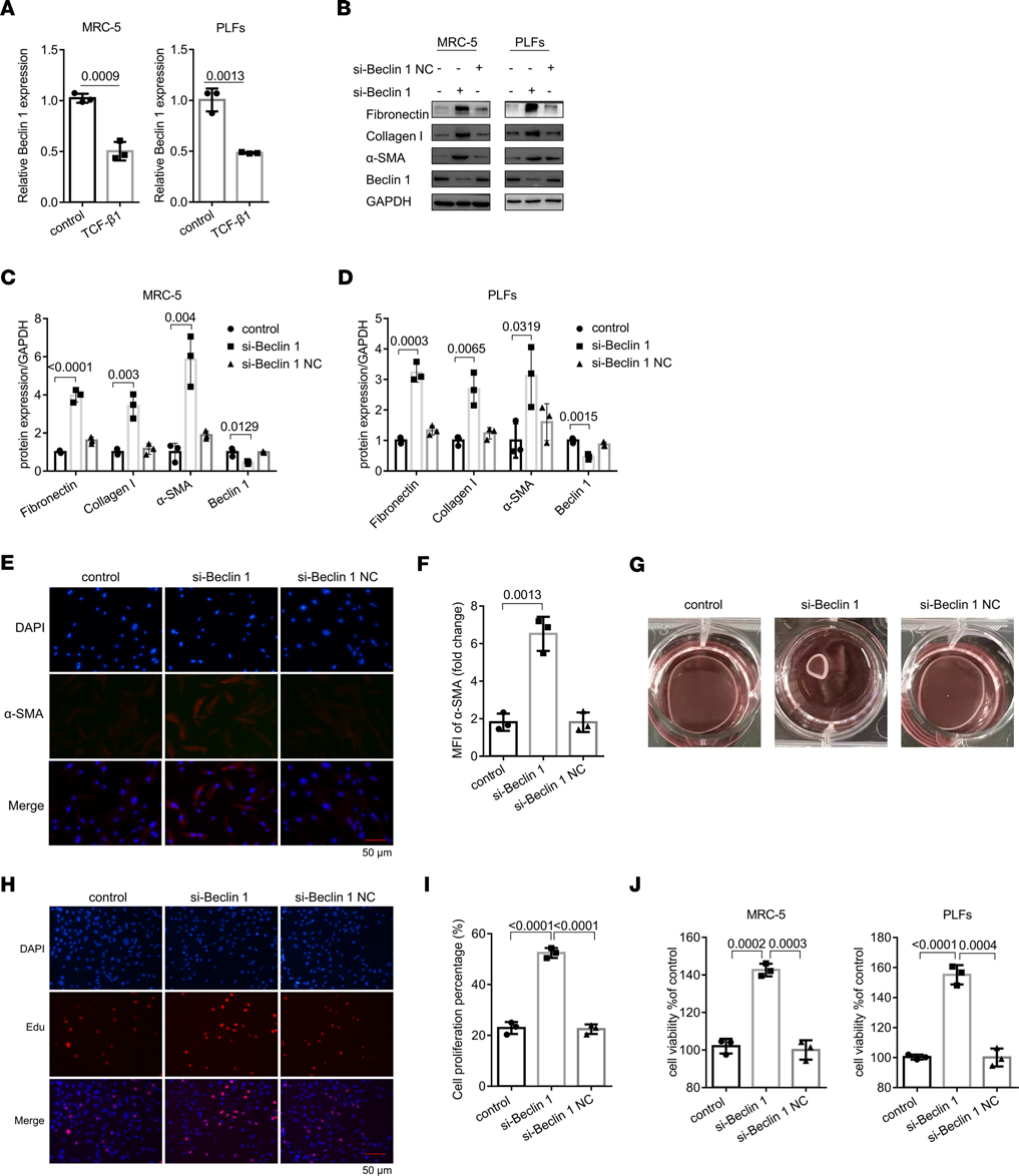
Beclin 1 is a functional downstream gene of UHRF1 and negatively regulates cell proliferation. (**A**) qRT-PCR analysis of beclin 1 expression in MRC-5 cells and PLFs after treatment with TGF-β1. Data are shown as the mean ± SEM (*n* = 3 in each group). (**B**–**D**) Western blot and corresponding densitometry analysis of fibronectin, collagen I, α-SMA, and beclin 1 in beclin 1 siRNA–treated MRC-5 cells and PLFs or those treated with its negative control (NC) siRNA. Data are shown as the mean ± SEM (*n* = 3 in each group). (**E**) Immunohistochemical staining of α-SMA in MRC-5 cells after beclin 1 siRNA or its negative control siRNA treatment. Red represents α-SMA; blue represents DAPI. (**F**) Mean fluorescence intensity of α-SMA in MRC-5 cells from the different groups. Data are shown as the mean ± SEM (*n* = 3 in each group). (**G**) Collagen gel contraction assay was performed to detect the effect of beclin 1 siRNA and its negative control siRNA on the contractility of fibroblasts. (**H** and **I**) Proliferation of MRC-5 cells transfected with beclin 1 siRNA and its negative control siRNA, as assessed by EdU assays. Data are shown as the mean ± SEM (*n* = 3 in each group). (**J**) CCK8 assays were performed to evaluate cell proliferative ability in fibroblasts. Data are shown as the mean ± SEM (*n* = 3 in each group). Scale bar: 50 μm (**E** and **H**). *P* values were from (**A**) a 2-tailed unpaired Student’s *t* test and (**C**, **D**, **F**, **I**, and **J**) a 1-way ANOVA post hoc test with Tukey’s correction.

Above, we have confirmed that beclin 1 might be a downstream gene of UHRF1; its expression accompanies cell autophagy and proliferation (16, 17). Additionally, we detected the downregulation of beclin 1 in TGF-β1–induced fibroblasts (Figure 5A). In addition, beclin 1 was decreased in silica- and BLM-treated lung tissues through immunofluorescence staining and qRT-PCR assays (Supplemental Figure 5, A and B). Next, we used siRNAs to knockdown beclin 1 expression in MRC-5 cells and PLFs and then detected the knockdown efficiency at mRNA and protein levels (Supplemental Figure 5C and Figure 5, B–D). Similarly, beclin 1 siRNA induced a robust increase of fibrotic marker protein levels and staining of α-SMA in lung fibroblasts (Figure 5, E and F, and Supplemental Figure 5D). Next, collagen gel contraction, EdU, and CCK8 assays showed that loss of beclin 1 promoted collagen contraction and increased the proliferation activity of cells (Figure 5, G–J). Besides, overexpression of P62 and downregulation of LC3B (the markers of autophagy) were also observed in beclin 1 siRNA–treated fibroblasts (Supplemental Figure 5, E–G). Similarly, our previous study demonstrated that beclin 1 facilitates PARK2 translocation to mitochondria, involving the mitophagy process in TGF-β1–stimulated fibroblasts (18).

**Figure 6:**
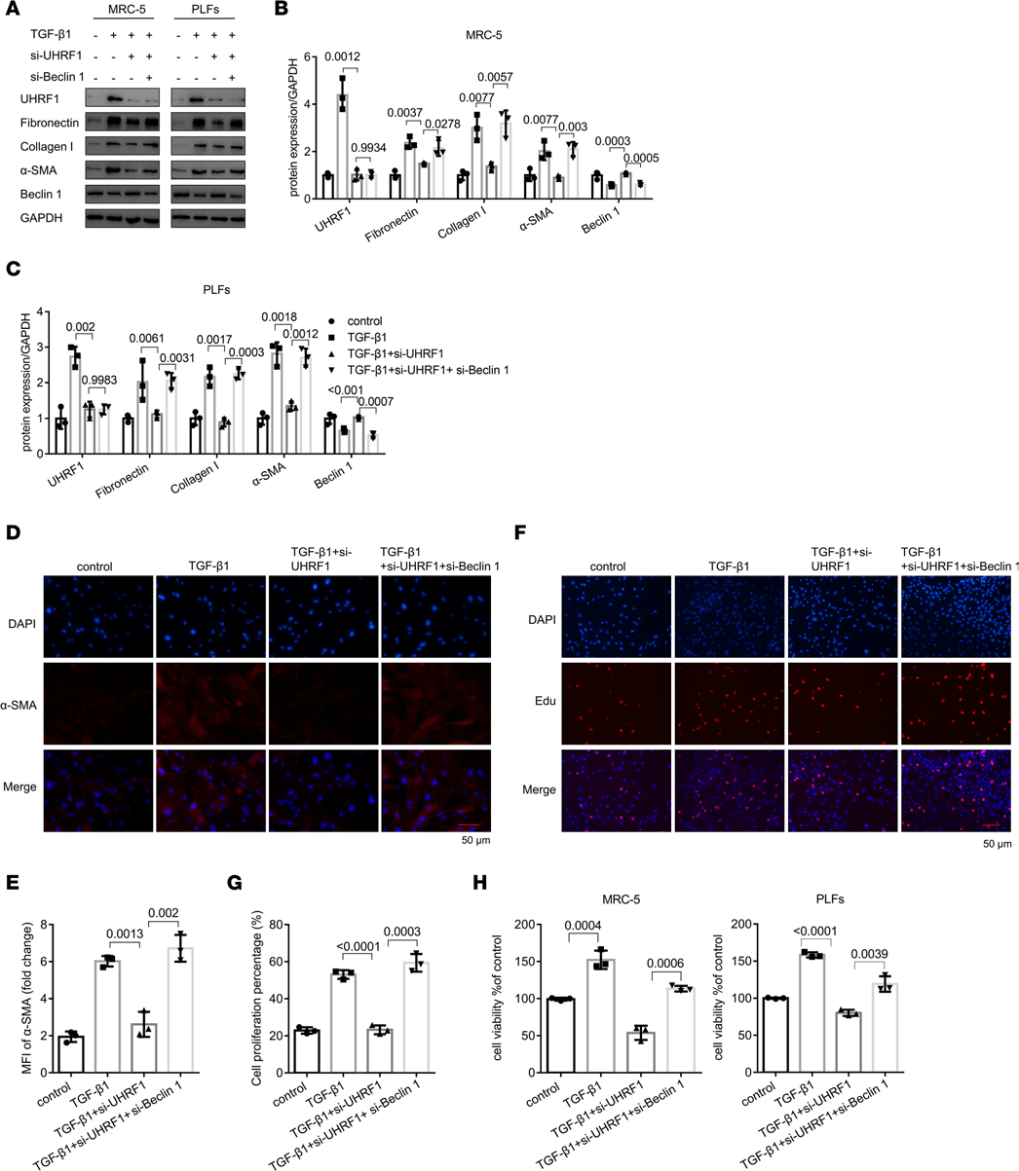
Loss and gain of function of beclin 1 reversed the effect of UHRF1 in fibroblasts. (**A**–**C**) Western blot and corresponding densitometry analysis of UHRF1, fibronectin, collagen I, α-SMA, and beclin 1 in the MRC-5 cells and PLFs for the indicated groups. Data are shown as the mean ± SEM (*n* = 3 in each group). (**D**) Immunohistochemical staining of α-SMA in MRC-5 cells for the indicated groups. α-SMA stained red; DAPI stained blue. (**E**) Mean fluorescence intensity of α-SMA in MRC-5 cells from the different groups. Data are shown as the mean ± SEM (*n* = 3 in each group). (**F** and **G**) Proliferation of MRC-5 cells transfected with different treatment, as assessed by EdU assays. Data are shown as the mean ± SEM (*n* = 3 in each group). (**H**) CCK8 assays were performed to evaluate ability of MRC-5 cells and PLFs to proliferate. Data are shown as the mean ± SEM (*n* = 3 in each group). Scale bar: 50 μm (**D** and **F**). (**B**, **C**, **E**, **G**, and **H**) *P* values were from a 1-way ANOVA post hoc test with Tukey’s correction.

To determine whether beclin 1 acts as a functional gene downstream of UHRF1, we simultaneously knocked down UHRF1 and beclin 1 in cells. Notably, low expression of UHRF1 could inhibit fibroblast activation, α-SMA expression, cell proliferation, and cell viability, whereas the effects were reversed by simultaneous knockdown of UHRF1 and beclin 1 (Figure 6 and Supplemental Figure 6, A and B). Furthermore, beclin 1 plasmid significantly reduced the effect of UHRF1 in fibroblasts (Supplemental Figure 6, C and D). These results demonstrated that beclin 1 is a functional target gene of UHRF1 that negatively regulates cell activation.

### In vivo functional validation of UHRF1 siRNA–loaded liposomes.

**Figure 7:**
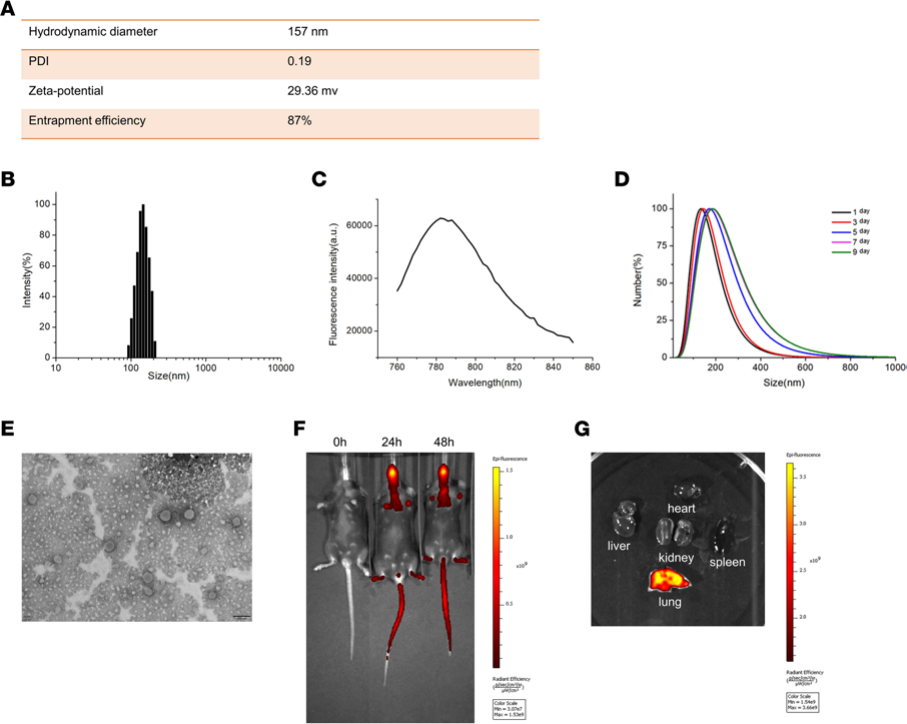
Characterization of UHRF1 siRNA–loaded liposomes. (**A**) The hydrodynamic diameter, PDI, ζ potential of the liposomes, and siRNA entrapment efficiency of UHRF1 siRNA–loaded liposomes. (**B**–**D**) Distribution of liposome size (**B**), fluorescence intensity (**C**), and stability of liposomes (**D**) loaded in UHRF1 siRNA–loaded liposomes. (**E**) Representative transmission electron microscopy image of UHRF1 siRNA–loaded liposomes. Scale bar: 200 nm. (**F**) Representative IVIS images of a mouse at different time points (0 hours, 24 hours and 48 hours) after the administration of DiR-labeled liposomes. (**G**) Ex vivo fluorescence images of major organs from mice.

Next, we sought to prepare for the delivery of UHRF1 siRNA to pulmonary fibrotic mice. As showed in Figure 7A, the prepared liposomes possessed a uniform size distribution of around 157 nm (PDI = 0.19) and displayed 87% entrapment efficiency for loading siRNA, with a ζ potential of 29.36 mv (Figure 7A). Next, we assessed the distribution of liposome size, fluorescence intensity, and the stability of loaded liposomes, as shown in Figure 7, B–D. Figure 7E shows a representative image, taken by transmission electron microscopy. The tail vein mainly accumulated fluorescence signal in the lung (Figure 7F). In addition, UHRF1 siRNA–loaded liposomes could be taken up by fibroblasts in a few hours and without cytotoxicity (Supplemental Figure 7, A and B). To further confirm this phenomenon, we collected organs, including the heart, liver, spleen, kidney, and lung, from mice 48 hours after tail vein injection of liposomes. As expected, ex vivo imaging only detected fluorescence in the lung and not in other organs (Figure 7G). To further investigate the liposome cellular localization in the fibrotic lung, we performed immunofluorescence staining assays by using mouse pulmonary tissue slides. Interestingly, liposomes were predominantly located in the fibrotic area of lung tissues and mainly overlapped with α-SMA^+^ cells, which indicated that the fibroblasts could efficiently absorb liposomes. However, liposomes could also target pulmonary epithelial cells, as shown by the costaining of E-cadherin and liposomes (Supplemental Figure 7C).

### Intravenous administration of UHRF1 siRNA–loaded liposomes alleviated pulmonary fibrosis in multiple experimental mouse models.

**Figure 8:**
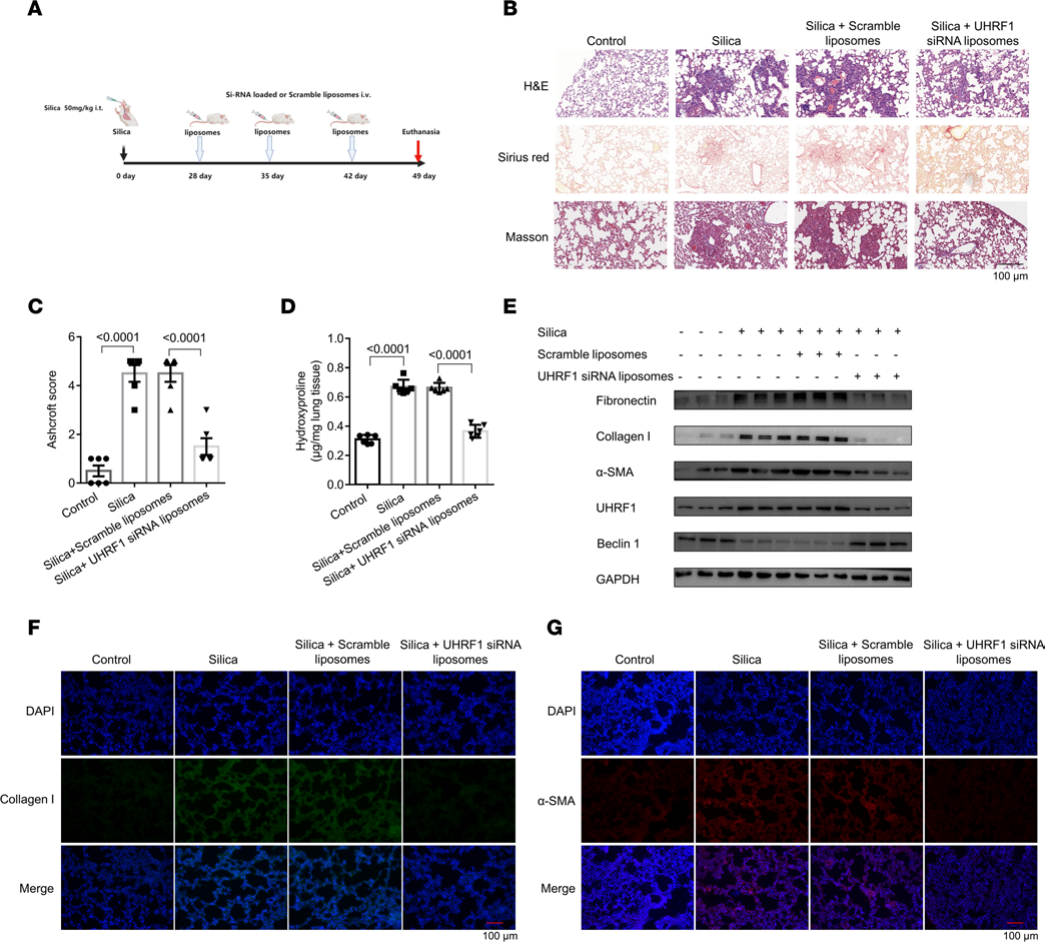
Administration of UHRF1 siRNA liposomes attenuates silica-induced pulmonary fibrosis in mice. (**A**) Strategy for UHRF1 siRNA–loaded liposome administration in the silica-induced pulmonary fibrosis mouse model. (**B**) H&E, Sirius red, and Masson’s trichrome staining assays were performed to measure the severity of the lung fibrosis. (**C**) Semiquantitative Ashcroft scores indicating the severity of fibrosis. Data are shown as the mean ± SEM (*n* = 6 in each group). (**D**) The hydroxyproline content in the lungs of mice for the different groups. Data are shown as the mean ± SEM (*n* = 6 in each group). (**E**) Western blot of UHRF1, fibronectin, collagen I, α-SMA, and beclin 1 in mouse lung tissues. (**F** and **G**) Immunohistochemical staining of collagen I and α-SMA in mouse lung tissues for the indicated groups. Collagen I stained blue; α-SMA stained red; DAPI stained blue. Scale bar: 100 μm (**B**, **F**, and **G**). (**C** and **D**) *P* values were from a 1-way ANOVA post hoc test with Tukey’s correction.

The therapeutic effects of UHRF1 siRNA–loaded liposomes were assessed in mice following silica (50 mg/kg) treatment for 28 days. Mice were administered with scrambled or UHRF1 siRNA–loaded liposomes by tail vein (dosage of siRNA, 1 mg/kg) on day 28, day 35, and day 42, respectively (Figure 8A). Administration of UHRF1 siRNA–loaded liposomes significantly attenuated silica-induced lung injury, as evidenced by the H&E, Sirius red, Masson’s trichrome staining and the Ashcroft score (Figure 8, B and C). Consistently, administration of UHRF1 siRNA–loaded liposomes decreased collagen content, as evidenced by hydroxyproline assays (Figure 8D), coupled with a marked reduction in the expression of fibrotic markers (Figure 8E). The body weight of mice indicated that the DiR-labeled liposomes could relieve weight loss caused by silica or BLM installation (Supplemental Figure 8A and Supplemental Figure 9A). Besides, an immunostaining assay and qRT-PCR assay showed that UHRF1, α-SMA, and collagen I in lung tissues from the UHRF1 siRNA–loaded liposome group were lower than in the scramble group, in contrast to the expression of beclin 1 (Figure 8, F and G, and Supplemental Figure 8, B–G).

**Figure 9:**
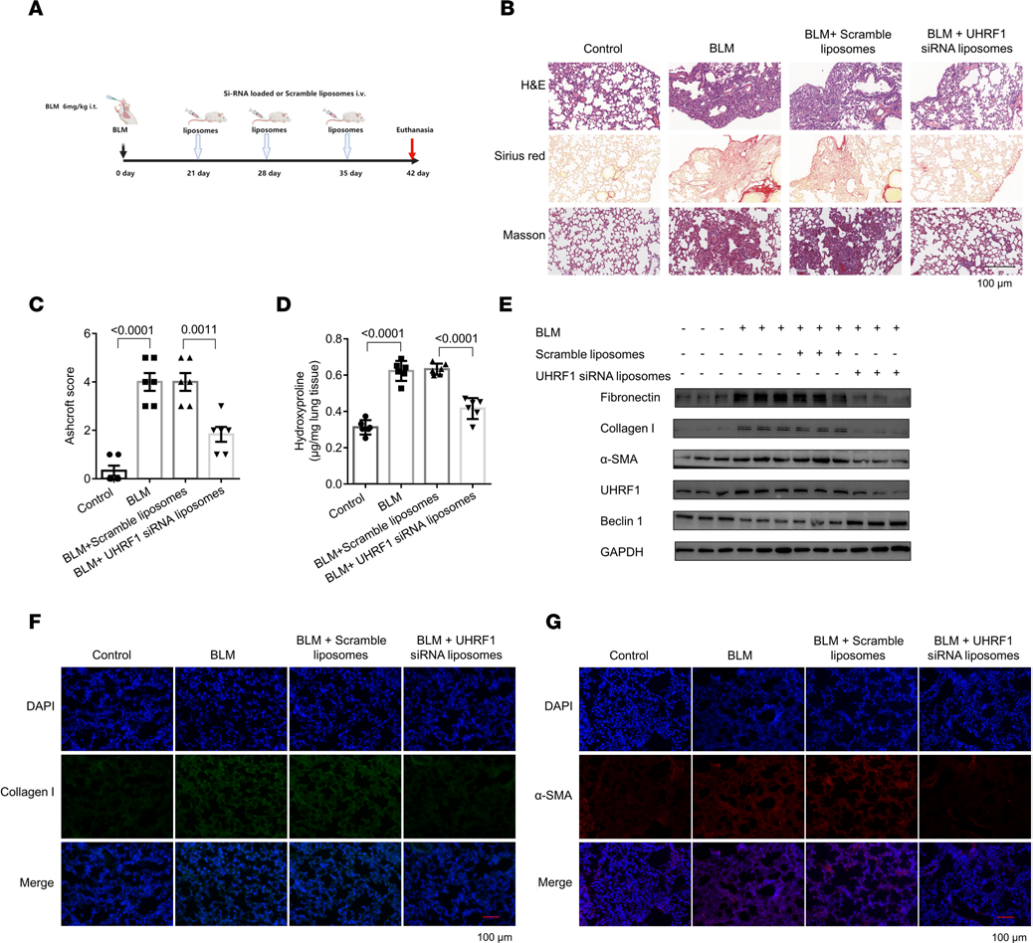
Administration of UHRF1 siRNA liposomes attenuates BLM-induced pulmonary fibrosis in mice. (**A**) Strategy for UHRF1 siRNA–loaded liposome administration in the BLM-induced pulmonary fibrosis mouse model. (**B**) H&E, Sirius red, and Masson’s trichrome staining assays were performed to measure the severity of fibrotic lesions. (**C**) The severity of fibrosis was evaluated by Ashcroft scores. Data are shown as the mean ± SEM (*n* = 6 in each group). (**D**) Lungs of mice following different treatments were analyzed for hydroxyproline content. Data are shown as the mean ± SEM (*n* = 6 in each group). (**E**) Western blot of UHRF1, fibrotic markers, and beclin 1 in mouse lung tissues in the different groups. (**F** and **G**) The expression of collagen I and α-SMA was detected by immunofluorescence staining in mouse lung tissues. Collagen I stained blue; α-SMA stained red; DAPI stained blue. Scale bar: 100 μm (**B**, **F**, and **G**). (**C** and **D**) *P* values were from a 1-way ANOVA post hoc test with Tukey’s correction.

We also made another pulmonary fibrosis model by administering BLM (6 mg/kg) (Figure 9A). Similarly, lung injury and Ashcroft scores as well as collagen content in lungs of the UHRF1 siRNA–loaded liposome group were decreased compared with those of the scrambled siRNA–loaded liposome group in BLM-treated mice, as shown by H&E, Sirius red, and Masson’s trichrome staining and hydroxyproline assays (Figure 9, B–D). Furthermore, the expression of fibrotic markers and UHRF1 were also decreased both at protein and mRNA levels after UHRF1 siRNA–loaded liposome treatment, in contrast to the expression of beclin 1 (Figure 9E and Supplemental Figure 9, B–E). In addition, immunostaining of α-SMA and collagen I, key fibrotic markers, as well as UHRF1 and beclin 1, was conducted in BLM-induced mouse lung sections. Following UHRF1 siRNA–loaded liposome administration, levels of α-SMA, collagen I, and UHRF1 were downregulated and levels of beclin 1 were upregulated (Figure 9, F and G, and Supplemental Figure 9, F and G). Together, these data suggested that the administration of UHRF1 siRNA liposomes attenuates pulmonary fibrosis in multiple experimental mouse models.

## Discussion

Pulmonary fibrosis is an interstitial lung disease, characterized by the deposition of excessive ECM, accumulation of proliferative fibroblasts, and the destruction of the parenchymal structure, which results in progressive lung function loss (12, 19). However, current treatment options are of limited benefit. This study investigated whether UHRF1 was overexpressed in lung fibroblasts stimulated by TGF-β1 and lungs that originated from fibrotic mice. Specifically, injection of liposomes carrying UHRF1 siRNA in the tail vein reversed silica- and BLM-induced pulmonary fibrosis. In general, TGF-β1 enhanced UHRF1 expression through the YAP/TEAD pathway. UHRF1 recognized and induced the methylated CpG sites in the beclin 1 promoter in a Dnmt1-dependent manner to repress beclin 1; this could be reversed by UHRF1 knockdown. A low level of UHRF1 or a high level of beclin 1, in turn, deactivated fibroblasts and blocked pulmonary fibrosis. Therefore, our results suggest that target therapy against UHRF1 could be a feasible clinical approach in the treatment of pulmonary fibrosis.

UHRF1 is a fundamental epigenetic regulator with multiple functions and has been recognized in many diseases (20). Previous studies have demonstrated that UHRF1 is involved in various processes, including histone modification and DNA methylation (21, 22). The maintenance of DNA methylation is of the most studied epigenetic functions of UHRF1. UHRF1 recruits Dnmt1 to maintain the methylation pattern via recognizing and binding hemimethylated DNA through its SRA domain (23). For example, Costello and colleagues reported that UHRF1 maintains KEAP1 promoter methylation, thus regulating the Nrf2 pathway in pancreatic cancer (24). Surprisingly, this study found that UHRF1 was overexpressed in TGF-β1–induced fibroblasts and fibrotic mouse lung tissues. Using UHRF1 siRNA could change UHRF1 downstream target gene beclin 1’s methylation pattern. We further conducted loss-of-function studies to examine the role of UHRF1 in lung fibroblasts. Indeed, UHRF1 knockdown reduced fibroblast activation, which was reversed by simultaneous knockdown of UHRF1 and beclin 1. These observations prompted us to attend to the role of UHRF1 in fibroblasts.

Next, we attempted to explore the underlying mechanism of UHRF1 in regulation of fibroblast activation. Previous studies have demonstrated that the TEAD protein family could bind to the UHRF1 promoter region, which indicates that UHRF1 could be regulated by the YAP/TEAD pathway (25). The YAP/TEAD pathway is a mechanical signal-related pathway that plays an essential role in vascular remodeling associated with cardiovascular disease (26). TGF-β1–induced YAP activation also contributes to fibroblast activation and the high level of fibrotic markers, such as fibronectin and α-SMA (27). As shown by Western blot and qRT-PCR analysis, silencing YAP and TEAD1/4 markedly inhibited the expression of UHRF1. Furthermore, ChIP experiments demonstrated the binding of YAP and TEAD1/4 to the UHRF1 promoter region. Therefore, these data indicated that UHRF1 was the downstream target of the YAP/TEAD pathway during fibroblast activation.

One of the most exciting discoveries in our results is that UHRF1 was noted to be involved in beclin 1 methylation during the fibrotic processes and, thus, is involved in regulation of fibroblast activation. Beclin 1, a key regulator of autophagy, is a central protein that assembles cofactors to trigger the autophagy protein cascade by forming a beclin 1–PIK3C3-PIK3R4 complex expression that accompanies cell proliferation (16). Previous studies have suggested that DNA methyltransferase could modulate the increase in methylation in the promoter region of beclin 1 in breast cancer (28). In the present study, we confirmed that the beclin 1 promoter in fibroblasts undergoes a DNA hypomethylation following UHRF1 knockdown, in which UHRF1 coregulates with Dnmt1 in methylation of CpG DNA within the beclin 1 promoter. After the downregulation of UHRF1 in fibroblasts, the expression of beclin 1 was upregulated and then attenuated pulmonary fibrosis. A recent study has shown that autophagic beclin 1 protein expression was decreased in idiopathic pulmonary fibrosis fibroblasts (29). Consistent with the previous studies, beclin 1 was downregulated in TGF-β1–treated fibroblasts in our study. We found that siRNA-medicated beclin 1 knockdown significantly promoted fibroblast activation.

Because pulmonary fibrosis is a chronic progressive disease of unknown cause, characterized by excessive collagen deposition and irreversible loss of lung function, there is no effective treatment that can cure pulmonary fibrosis (30). Nintedanib and pirfenidone are approved to treat pulmonary fibrosis, but neither drug cures the disease or improves lung function (31). This pulmonary fibrosis treatment background supports the ongoing critical need for drug discovery efforts. Over the past few decades, nanoparticle-based drug delivery platforms have been considered available vehicles to overcome pharmacokinetic limitations compared with conventional drug formulations (32). Moreover, lipid-based carriers (e.g., siRNAs, mRNAs, DNA) are safer compared with other delivery vectors (e.g., adeno-associated virus, adenovirus, lentivirus) and participate in the treatment of various diseases (33, 34). Previous studies have illustrated that liposome loading with siRNA and drugs has been established as an essential delivery system due to its effectiveness and safety in providing the treatment of pulmonary fibrosis in clinical settings (35, 36). Consistent with these studies, our results showed that liposomes carrying UHRF1 siRNA could efficiently reverse mouse pulmonary fibrosis in vivo. Notably, the liposomes could focally target the fibrotic area in the lung with high efficiency via vein tail injection. Taken together, liposomal-based UHRF1 siRNA provided a practical, available, and safe therapeutic approach for silica- and BLM-induced pulmonary fibrosis.

We demonstrated that UHRF1 was activated in silica- and BLM-induced fibrotic lung tissues and TGF-β1–stimulated fibroblasts. Liposome loading with UHRF1 siRNA protected lungs from injuries induced by silica and BLM. Mechanistically, UHRF1 inactivated beclin 1 via induction of de novo methylation of beclin 1 promoter in a Dnmt1-dependent manner, leading consequently to the activation of fibroblasts. In summary, our results suggest that liposomal-based UHRF1 siRNA is a potential therapy against pulmonary fibrosis.

## Methods

### Cell culture and treatment.

The human fibroblasts (MRC-5 cells) and mouse fibroblasts (NIH/3T3) were purchased from ATCC. The PLFs were isolated from the lung of the C57BL/6 mouse. MRC-5 cells were maintained in Minimum Essential Medium (Life Technologies/Gibco), and NIH/3T3 cells and PLFs were maintained in DMEM (Life Technologies/Gibco), which contained 10% fetal bovine serum (BISH1475, Biological Industries) and antibiotics (penicillin and streptomycin, Life Technologies/Gibco). All the cells were cultured at 37°C with 5% CO_2_. Cells were treated with 5 ng/mL recombinant TGF-β1 (Peprotech) for 48 hours to induce fibroblast activation. To inhibit UHRF1 expression, fibroblasts were treated with 15 μM NSC232003 for 4 hours after TGF-β1 treatment.

UHRF1 siRNA, beclin 1 siRNA, and control siRNA were synthesized by GenePharm. UHRF1 plasmid and beclin 1 plasmid were synthesized by Genery. Cells were transfected using riboFECTCP Reagent (Ribobio) according to the manufacturer’s protocol. The UHRF1 siRNA and beclin 1 siRNA sequences are shown in Supplemental Table 1. The UHRF1 inhibitor NSC232003 was phased from MedChemExpress.

### Preparation and characterization of UHRF1 siRNA–loaded liposomes.

Liposomes were used as carriers to encapsulate siRNA. The siRNA was dissolved in citrate buffer (10 mM, pH = 3) and rapidly mixed with a lipid mixture by vortexing. The lipid mixture was composed of C12-200, cholesterol, DSPC, and mPEG-DMG dissolved in ethanol at a molar ratio of 50:38.5:10:1.5. The next step was to exclude unentrapped siRNA by ultrafiltration centrifugation. Lastly, the siRNA liposomes were diluted in phosphate-buffered saline. The characteristics of liposomes (hydrodynamic diameter, ζ potential, morphology, and stability) are listed in Figure 6. To detect the biodistribution of the liposomes, mice received a single tail vein injection with 1 mg/kg DiR-labeled liposomes. The mice were then anesthetized and imaged by an imaging system (PerkinElmer, IVIS Spectrum).

### BLM induction of pulmonary fibrosis.

C57BL/6 mice (5–6 weeks old) were randomly divided into 4 groups (*n* = 6 each group): a saline group, a BLM-induced pulmonary fibrotic group, a BLM plus UHRF1 siRNA–loaded liposome group, and a BLM plus scrambled siRNA–loaded liposome group. On day 0, mice received a single intratracheal instillation with 6 mg/kg BLM (MKBio Technology Co. Ltd.). Sterile saline was administered by intratracheal instillation as a control. UHRF1 or scrambled siRNA–loaded liposomes (1 mg/kg, Ruixi Biological Technology Co. Ltd) were injected into the intervention animals via tail vein on days 21, 28, and 35 after BLM injection. On day 42, mice were euthanized, and lung tissues were collected for analysis of pulmonary fibrosis.

### Silica induction of pulmonary fibrosis.

C57BL/6 mice (5–6 weeks old) were randomly divided into 4 groups (*n* = 6 in each group): a saline group, a silica-induced pulmonary fibrotic group, silica plus UHRF1 siRNA–loaded liposome group, and silica plus scrambled siRNA–loaded liposome group. On day 0, mice received a single intratracheal instillation with 50 mg/kg silica particles (size distribution, 99% between 0.5 and 10 μm, 80% between 1 and 5 μm; average particle diameter, 1.7 μm; Sigma-Aldrich) dissolved in 0.05 mL sterile saline. Sterile saline was administered by intratracheal instillation as a control. After silica treatment for 28 days, the mice in the liposome treatment groups received liposomes by tail vein on days 28, 35, and 42. Then, the mice were sacrificed, and the lung tissues were stored at –80°C for further analysis.

### Histopathology.

The fresh mouse lung tissues were collected and soaked in fresh 4% neutral-buffered paraformaldehyde overnight. After being embedded in paraffin, lung tissues were cut into 5 μm thick slices. Then, the lung tissue sections were used for H&E, Masson’s trichrome, and Sirius red staining to assess the degree of fibrosis. The fibrosis of lung tissues was evaluated based on the change of alveolar wall thickening, inflammatory lesions, and cellular proliferation.

### IHC.

Paraffin-embedded human lung tissue sections were subjected to immunostaining. After deparaffinization and antigen retrieval, the lung tissue sections were incubated overnight with UHRF1 antibodies, followed by scanning with a Pannoramic Scanning Electron Microscope (3DHISTECH).

### Hydroxyproline content assay.

The hydroxyproline content of the lung tissues was determined by the hydroxyproline content assay (Jiancheng Bioengineering Institute, A030-2). According to the manufacturer’s protocol, the levels of hydroxyproline content were determined at 550 nm and expressed as μg per mg of lung tissues.

### Immunofluorescent staining.

Adherent cells were fixed with 4% paraformaldehyde for 30 minutes, washed with PBS, and blocked with 10% BSA for 1 hour at room temperature. Then, cells were stained with a primary antibody against α-SMA (Abcam, ab32575, 1:200), an antibody against collagen I (ABclonal, A1352), an antibody against UHRF1 (Santa Cruz, H-8, sc-373750, 1:100), and antibody against beclin 1 (ABclonal, A7353, 1:200) overnight at 4°C and incubated with Cy3-conjugated or FITC-conjugated goat or mouse anti-rabbit antibody (1:200, Beyotime Institute of Biotechnology) for 1 hour. The nuclear DNA was then stained with DAPI. Fluorescence images were acquired with the fluorescence microscope (Olympus).

### Western blot and antibodies.

Lung tissues and cultured cells were homogenized in T-PER Tissue Protein Extraction Reagent (Thermo Scientific), and RIPA lysis buffer was added with phenylmethylsulfonyl fluoride for extraction of total proteins (Beyotime Institute of Biotechnology, Shanghai, China; PMSF, Sigma-Aldrich). A total of 80 μg of protein extracts were separated on 10% polyacrylamide gels (Sigma-Aldrich) and transferred onto polyvinylidene difluoride membranes (0.2 μm, Immobilon, ISEQ00010). Protein bands were next incubated with indicated primary antibodies for analysis of protein levels as described previously (37).

The following primary antibodies were used: antibody against fibronectin (Abcam, ab45688); antibody against collagen I (ABclonal, A1352); antibody against α-SMA (Abcam, ab32575); antibody against UHRF1 (Santa Cruz, H-8, sc-373750); antibody against beclin 1 (ABclonal, A7353); antibody against Dnmt1 (Santa Cruz, sc-271729); antibody against TEAD1 (ABclonal, A13366); antibody against TEAD4 (ABclonal, A4151); antibody against YAP (Proteintech, 13584-1-AP); and antibody against GAPDH (ABclonal, AC002).

### Quantitative RT‑PCR.

Quantitative RT‑PCR was performed using the HiScript II Q Select RT Supermix (Vazyme Biotech Co.) and the AceQ qPCR SYBR Green Master Mix kit (Vazyme Biotech Co. Ltd.). Fold changes in each target gene expression level were analyzed using the 2-^ΔΔCt^ method, and the relative expression of the target gene was normalized by GAPDH. The primers used in our study are shown in Supplemental Table 1.

### EdU incorporation assay.

According to the manufacturer’s instructions, cell proliferation was detected using the Cell-Light EdU Apollo 567 Kit (RiBoBio). After the indicated treatment, cells were plated in 96-well plates and incubated with 50 μM Edu for 2 hours. Cells were then fixed with 4% paraformaldehyde and stained with Apollo Dye Solution for labeling the proliferating cells. Cell nuclei were mounted with 1× Hoechst 33342. Immunofluorescence was acquired with the fluorescence microscope (Olympus).

### ChIP-qPCR.

ChIP assays were performed using the SimpleChIP Enzymatic Chromatin IP Kit (CST, 9003). Briefly, cells were harvested and cross-linked with 1% formaldehyde, followed by the addition of 10× glycine for 5 minutes at room temperature. Then, the cells were washed and collected in centrifuge tubes, after sonicating and immunoprecipitating with IgG, anti-UHRF1, anti-TEAD1, anti-TEAD4, anti-YAP, anti-Dnmt1, and anti-Histone antibodies at 4°C overnight. Then, the immunoprecipitates were eluted and reverse cross-linked, followed by purification of DNA fragments for PCR amplification. Primer sequences are listed in Supplemental Table 2.

### Methylation-specific PCR.

The EpiArt DNA Methylation Bisulfite Kit (Vazyme Biotech Co. Ltd., EM101-01) was used to detect the methylation level of the beclin 1 promoter region. The genomic DNA extraction kit (Beyotime Institute of Biotechnology, D0061) was used to extract genomic DNA. The PCR primers for methylation and unmethylation of the beclin 1 gene are listed in Supplemental Table 3. PCR reactions were carried out by agarose gel electrophoresis, and the gel was directly visualized with the Doc XR + gel imaging system (Bio-Rad).

### Collagen gel contraction assay.

Collagen gels were prepared using 1–2 mg/mL rat tail collagen I (Xinyou Biotechnology Co. Ltd., 200110) that was neutralized with 1 M NaOH and supplemented with 10× PBS. NIH/3T3 cells were seeded in collagen gel (1–2 mg/mL) solution and incubated at room temperature for 20 minutes until the gel was allowed to solidify. The collagen gel was then incubated in 10% FBS cell culture medium at 37°C in 5% CO_2_ environment for 24 hours and treated as indicated. Gels were imaged at the end of the experiment.

### Statistics.

Comparisons between groups were undertaken using Graph Pad Prism (version 6.01) software. Statistical analysis was performed using 2-tailed Student’s *t* test (between 2 groups) or 1-way ANOVA followed by Tukey’s multiple comparisons test (more than 2 groups). A post hoc test with Tukey’s correction was applied to multiple comparisons. The data were presented as mean ± SD unless otherwise specified. In all cases, *P* values of less than 0.05 were considered statistically significant. Specifically, biologic replicates were performed in cell-based assays from different experiments. Animal experiments findings were repeated in multiple experiments.

### Study approval.

All animal experiments were approved by Nanjing Medical University Ethics Committee. Six-week-old male C57BL/6 mice were purchased from the Animal Center of Nanjing Medical University and housed in specific pathogen–free conditions. Lung tissues were collected from patients with Idiopathic pulmonary fibrosis and silicosis and participants with normal lungs at Nanjing Drum Tower Hospital (Nanjing, China) after informed consent was received. The Human Assurance Committee approved the Nanjing Medical University experiments.

## Author contributions

DC conceptualized the study, provided methodology, and wrote the original draft of the manuscript. YW acquired data and provided methodology. ZL provided methodology and software. HX provided visualization and interpretation of data. WS and SZ curated data and provided investigation. SX reviewed and edited the manuscript. YL and CN supervised the study and reviewed and edited the manuscript.

## Supplementary Material

Supplemental data

## Version 1 09/27/2022

In-Press Preview

## Version 2 11/22/2022

Electronic publication
