# Supplementary material for: Liposomal UHRF1 siRNA shows lung fibrosis treatment potential through regulation of fibroblast activation
Source: JCI Insight. 2022 Nov 22;7(22):e162831. doi: 10.1172/jci.insight.162831 (PMC9746815; doi:10.1172/jci.insight.162831)
Supplement: Supplemental data [file jciinsight-7-162831-s090.pdf]

Revised supplementary materials

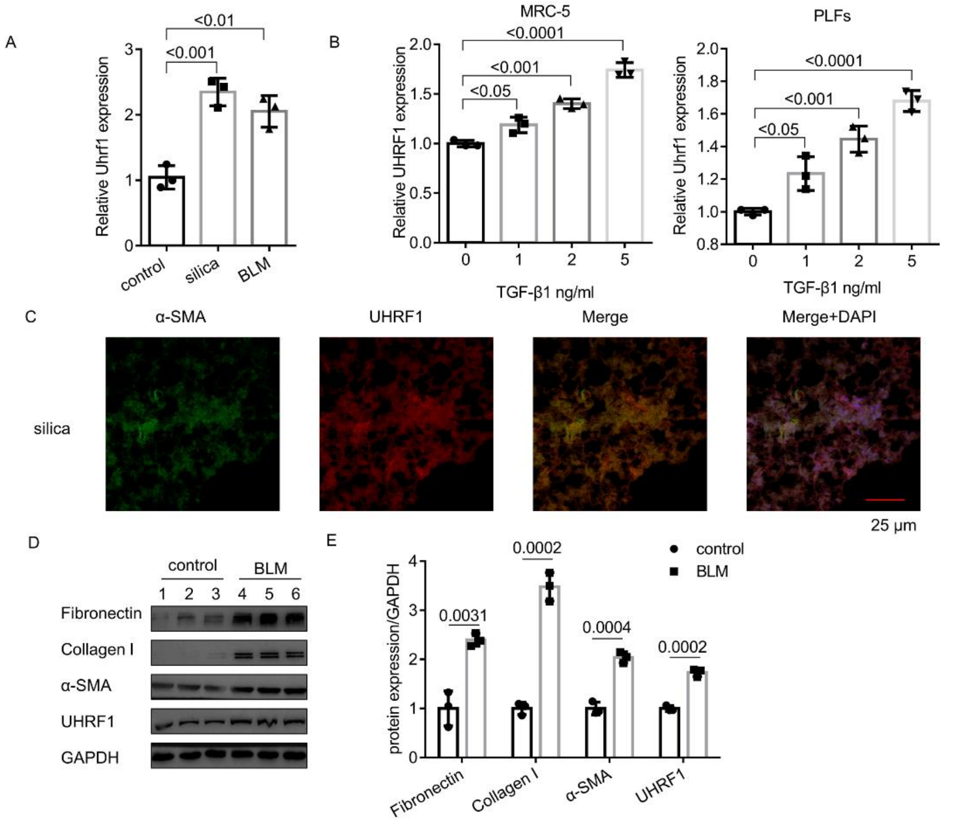

Fig. S1. UHRF1 is overexpressed in TGF-β1-stimulated fibroblasts and fibrotic lungs.

(A) qRT-PCR analysis of UHRF1 expression in silica or BLM-treated mouse lung tissues. Data are shown as mean ± SEM (n = 3 in each group). (B) qRT-PCR analysis of UHRF1 expression in MRC-5 and PLFs treated with TGF-β1. Data are shown as

mean ± SEM (n = 3 in each group). (C) Representative results for co-immunostaining of UHRF1 and α-SMA in the lung sections treatment with silica. Red represents UHRF1; Green represents α-SMA; blue represents nuclear DNA staining by DAPI. (D-E) Western blot and densitometric analysis of the protein of Fibronectin, Collagen I, α-SMA, and UHRF1 in saline or BLM treated mouse lung tissues. Data are shown as mean ± SEM (n = 3 in each group). (A-B) *P* values were from a one-way ANOVA post hoc test with Tukey correction. (E) *P* values were from a two-tailed unpaired Student *t*-test.

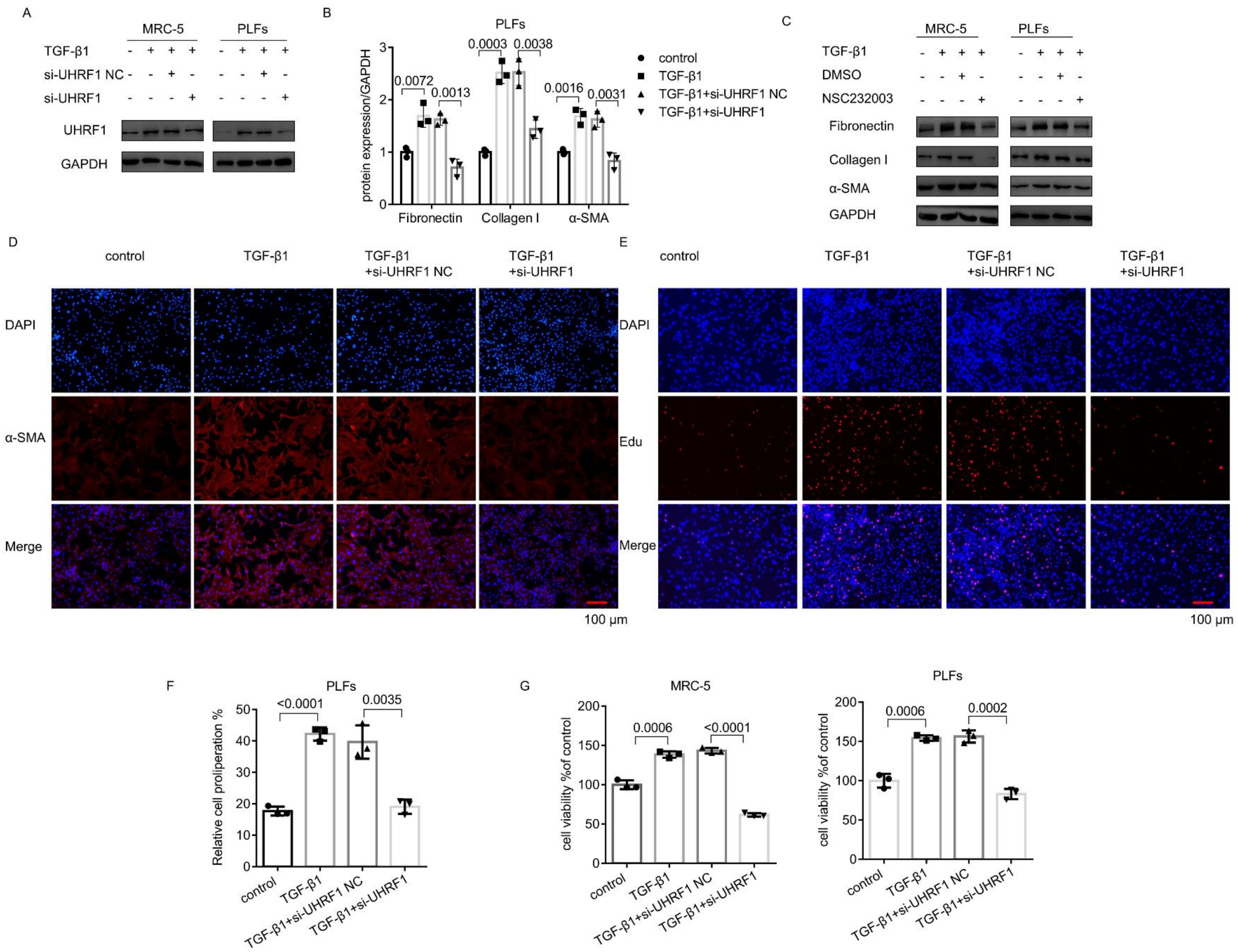

Fig. S2. UHRF1 regulates TGF-β1-induced lung fibroblast proliferation.

(A) Western blot of UHRF1 in MRC-5 and PLFs transfected with UHRF1 siRNA, and its negative control (NC) siRNA was then treated with 5 ng/Ml TGF-β1 for 48 h. (B) Densitometry analysis of Fibronectin, Collagen I, α-SMA in PLFs transfected with UHRF1 siRNA, and its negative control (NC) siRNA then treated with 5 ng/Ml TGF-β1 for 48 h. Data are shown as mean ± SEM (n = 3 in each group). (C) Western blot of Fibronectin, Collagen I, α-SMA in MRC-5 and PLFs treated with UHRF1 inhibitor NSC232003, and its negative control (NC) then treated with 5 ng/Ml TGF-β1 for 48 h. (D) The expression of α-SMA was detected by immunofluorescence staining in PLFs cells transfected with UHRF1 siRNA, and its negative control (NC) siRNA was then treated with 5 ng/Ml TGF-β1 for 48 h. α-SMA stained red; DAPI stained blue. (E-F) Cell proliferation of PLFs transfected with UHRF1 siRNA and its negative control (NC) siRNA, assessed by EdU assays. Data are shown as mean ± SEM (n = 3 in each group). (G) CCK8 assays were performed to evaluate cell proliferative ability in MRC-5 and PLFs transfected with UHRF1 siRNA, and its negative control (NC) siRNA was then treated with 5 ng/Ml TGF-β1 for 48 h. Data are shown as mean ± SEM (n = 3 in each group). (B and F-G) *P* values were from a one-way ANOVA post hoc test with Tukey correction.

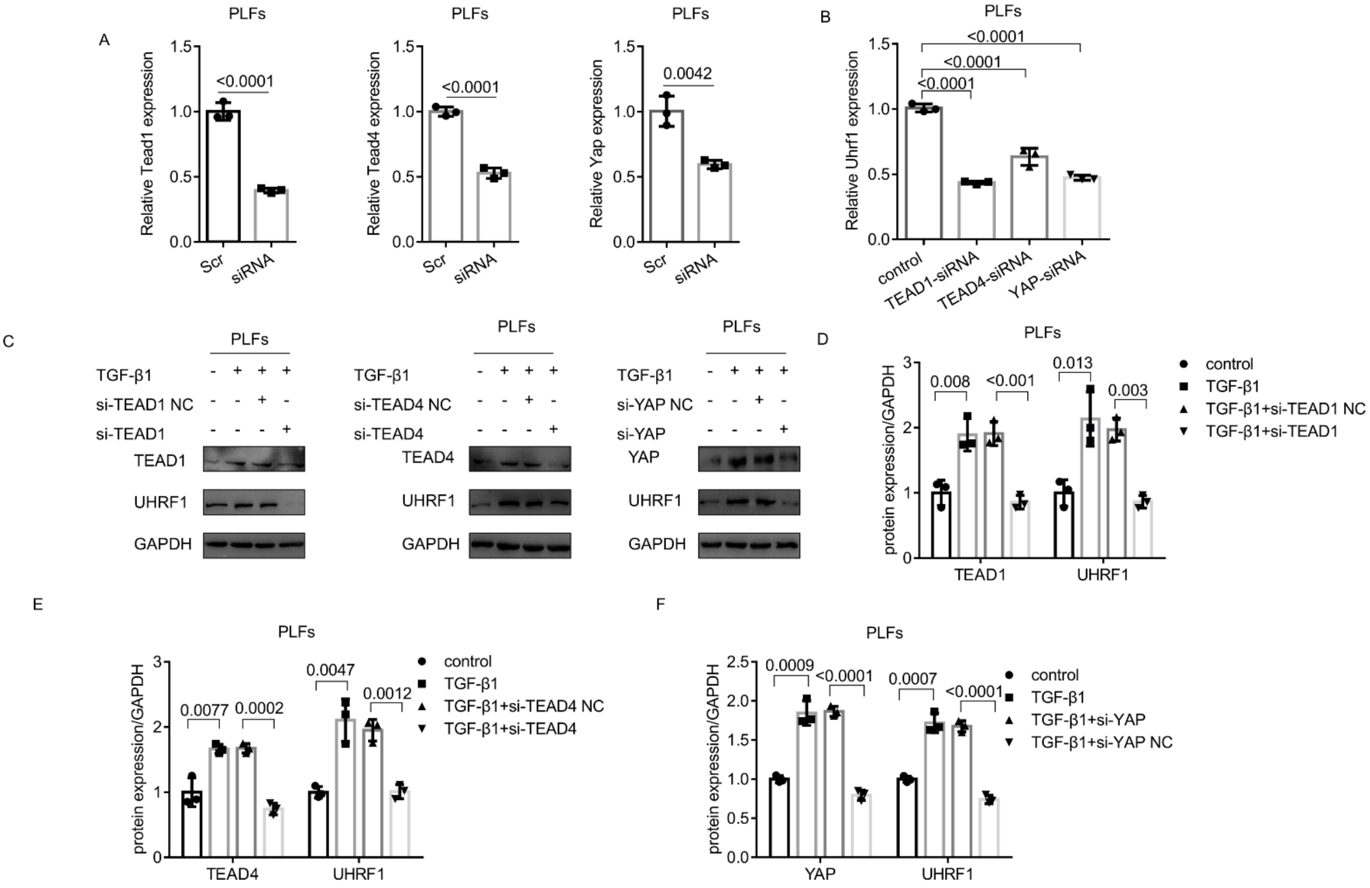

Fig. S3. YAP-TEAD pathway contributes to the expression of UHRF1 in fibroblast activation.

(A) Target siRNA transfection significantly decreased the expression of TEAD1, TEAD4, and YAP in PLFs. Data are shown as mean ± SEM (n = 3 in each group). (B) Qrt-PCR detection of UHRF1 expression in PLFs after transfected with TEAD1, TEAD4, and YAP siRNA. Data are shown as mean ± SEM (n = 3 in each group). (C-F) Western blot and corresponding densitometry analysis of TEAD1, TEAD4,

YAP and UHRF1 in PLFs transfected with TEAD1, TEAD4, and YAP siRNA, and their negative control (NC) siRNA then treated with 5 ng/Ml TGF-β1 for 48 h. Data are shown as mean ± SEM (n = 3 in each group). (A) *P* values were from a two-tailed unpaired Student *t*-test. (B and D-F) *P* values were from a one-way ANOVA post hoc test with Tukey correction.

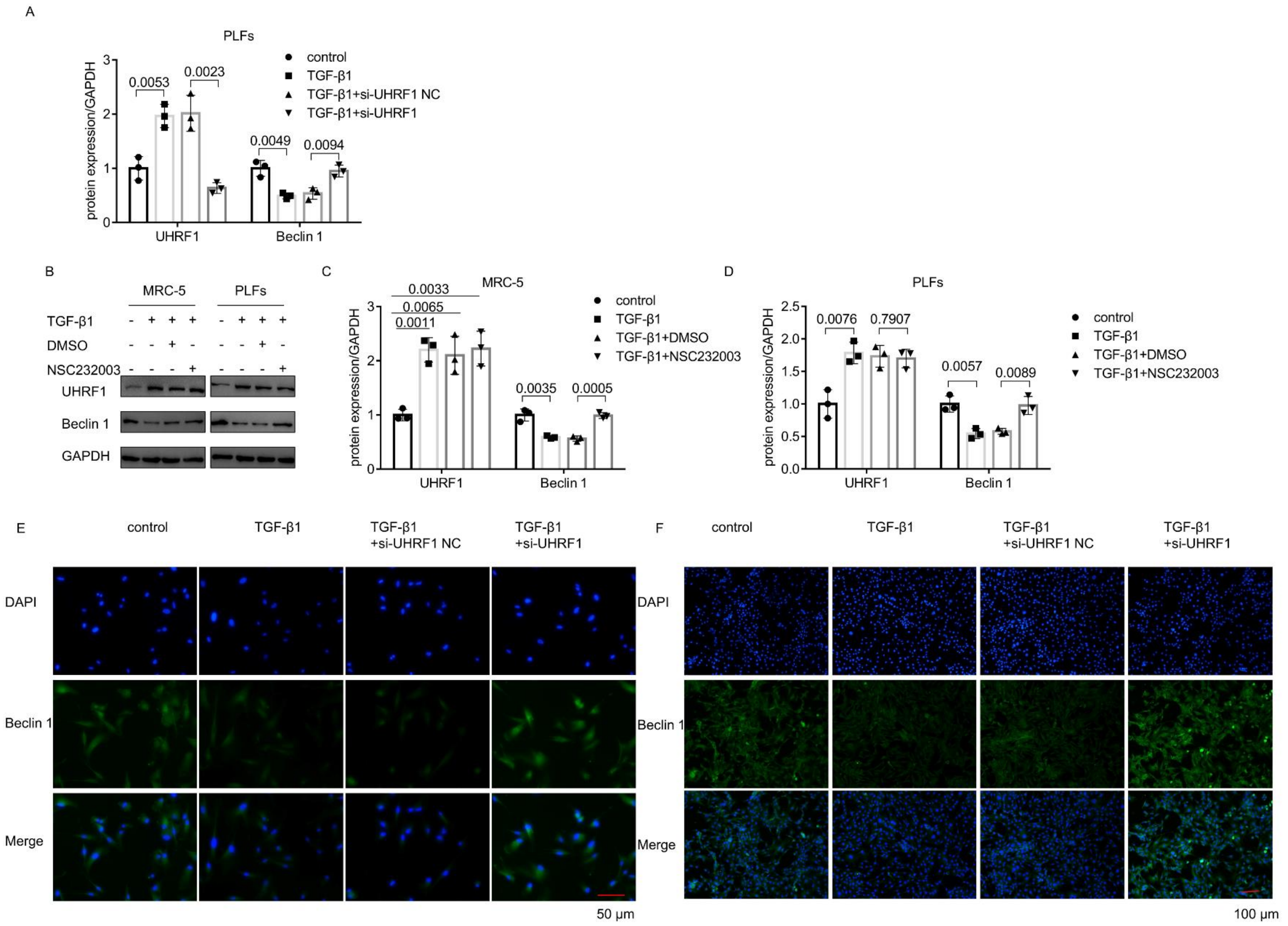

Fig. S4. UHRF1 epigenetically mediates Beclin 1 methylation in lung fibroblasts

(A) Corresponding densitometry analysis of UHRF1 and Beclin 1 in PLFs

transfected with UHRF1 siRNA, and its negative control (NC) siRNA was then treated with 5 ng/mL TGF-β1 for 48 h. Data are shown as mean ± SEM (n = 3 in each group). (B-D) Western blot and corresponding densitometry analysis of UHRF1 and Beclin 1 in MRC-5 cells and PLFs treated with UHRF1 inhibitor NSC2320003 and its negative control (NC). Data are shown as mean ± SEM (n = 3 in each group). (E-F) The expression of Beclin 1 was detected by immunofluorescence staining in MRC-5 cells and PLFs cells transfected with UHRF1 siRNA, and its negative control (NC) siRNA was then treated with 5 ng/mL TGF-β1 for 48 h. Beclin 1 stained green; DAPI stained blue. (A and C-D) *P* values were from a one-way ANOVA post hoc test with Tukey correction.

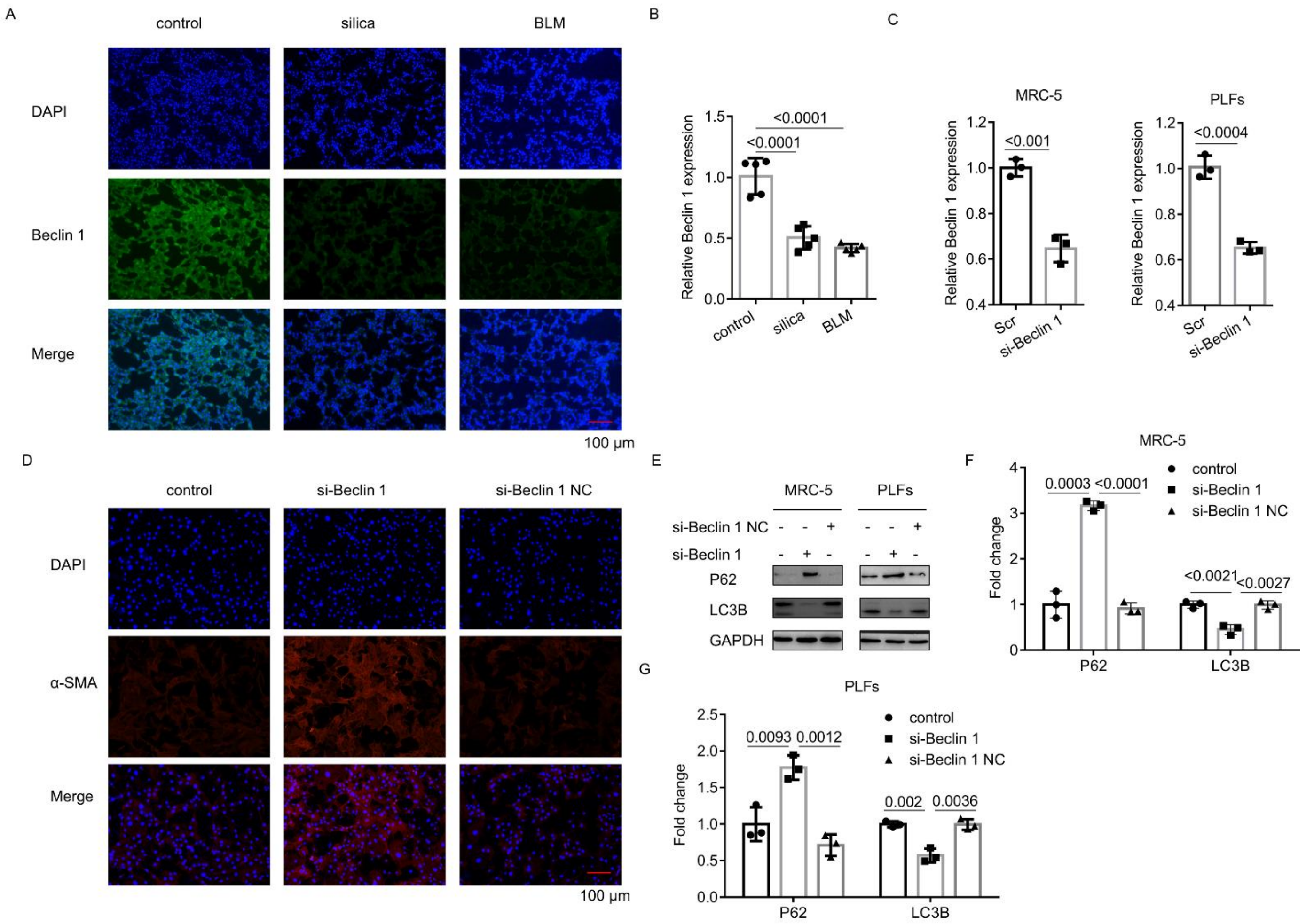

Fig. S5. Beclin 1 is a functional downstream gene of UHRF1 and negatively regulates cell proliferation.

(A) Immunofluorescence staining of Beclin 1 in silica or BLM-treated lung tissues. Beclin 1 stained green; DAPI stained blue. (B) qRT- PCR detection of Beclin 1 expression in silica or BLM treated lung tissues. Data are shown as mean ± SEM (n = 5 in each group). (C) qRT- PCR analysis of Beclin 1 expression in MRC-5 cells and PLFs after transfected with Beclin 1 siRNA or its negative control (NC) siRNA. Data are shown as mean ± SEM (n = 3 in each group). (D) The expression of α-

SMA was detected by immunofluorescence staining in PLFs cells after being transfected with Beclin 1 siRNA or its negative control (NC) siRNA. α-SMA stained red; DAPI stained blue. (E-G) Western blot and corresponding densitometry analysis of P62 and LC3B in MRC-5 cells and PLFs after being transfected with Beclin 1 siRNA or its negative control (NC) siRNA. Data are shown as mean ± SEM (n = 3 in each group). (C) *P* values were from a two-tailed unpaired Student *t*-test. (B and F-G) *P* values were from a one-way ANOVA post hoc test with Tukey correction.

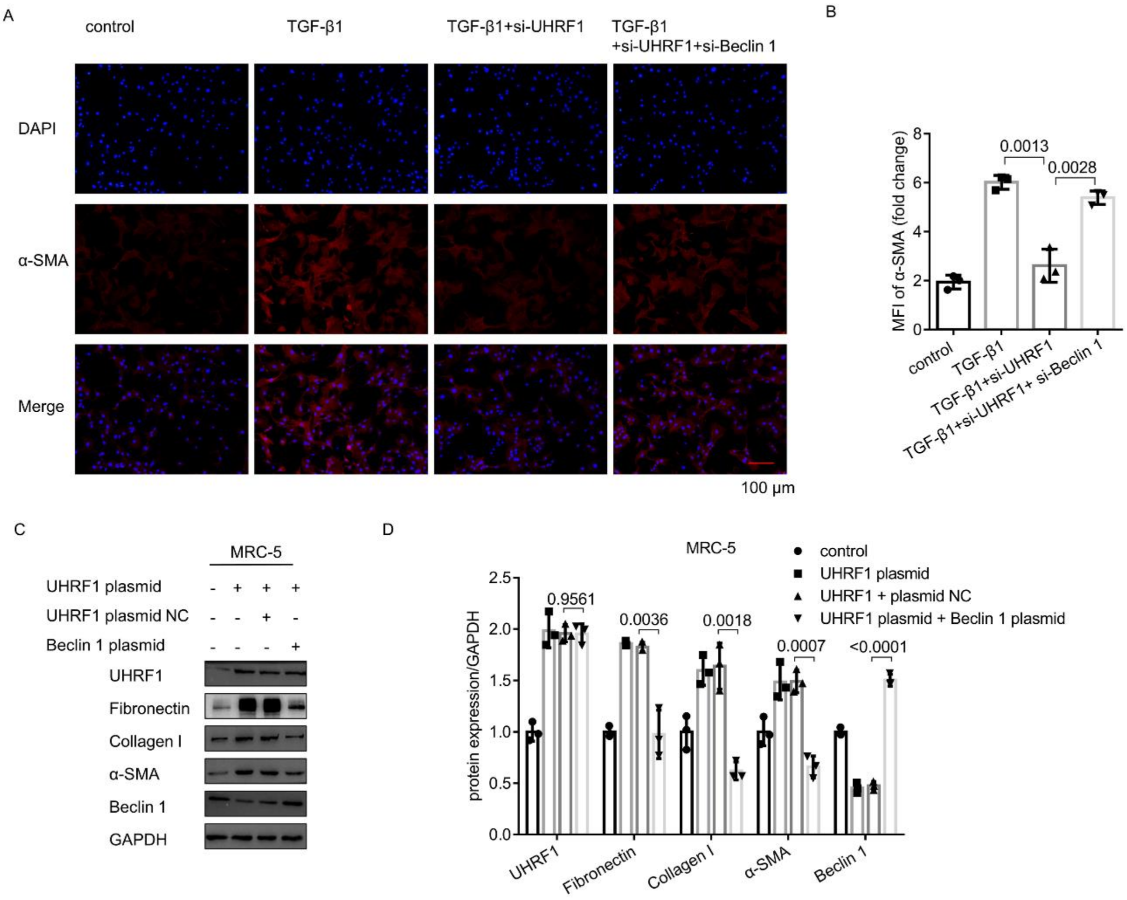

Fig. S6. Loss-and gain-of-functions of Beclin 1 reversed the effect of UHRF1 in fibroblasts.

(A) The expression of α-SMA was detected by immunofluorescence staining in PLFs cells for the indicated groups. α-SMA stained red; DAPI stained blue. (B) Mean fluorescence intensity of α-SMA in PLFs from the different groups. Data are shown as mean ± SEM (n = 3 in each group). (C-D) Western blot and corresponding densitometry analysis of UHRF1, Fibronectin, Collagen I, α-SMA and Beclin 1 in MRC-5 cells treated with different treatment. Data are shown as mean ± SEM (n = 3 in each group). (B and D) *P* values were from a one-way ANOVA post hoc test with Tukey correction.

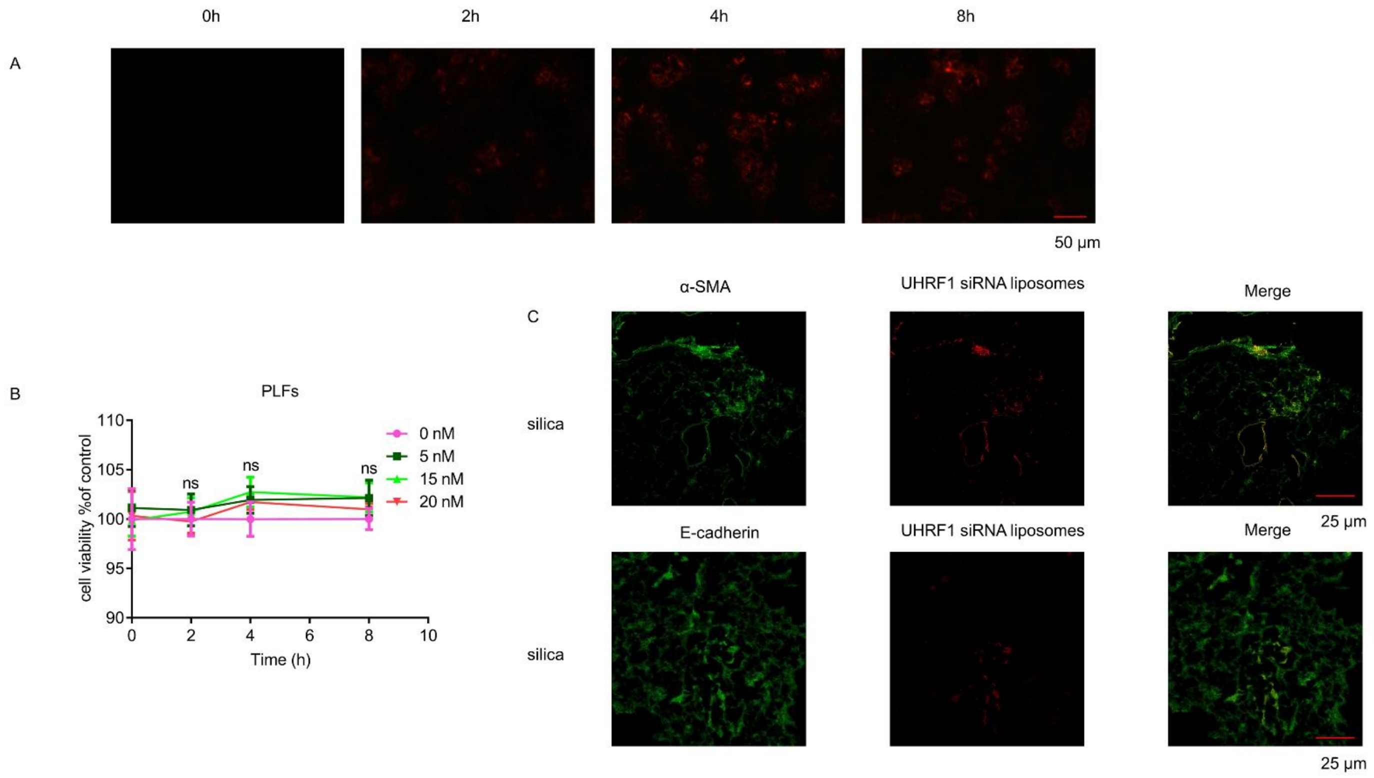

Fig. S7. The characterization of UHRF1 siRNA-loaded liposomes.

(A) Immunofluorescence of UHRF1 siRNA-loaded liposomes in fibroblasts, DiR-labeled liposomes marked red. (B) CCK8 assays were performed to evaluate the effect of UHRF1 siRNA-loaded liposomes on fibroblasts. (C) Representative results for co-immunostaining of liposomes and α-SMA (the marker of fibroblast) or E-cadherin (the marker of epithelial cells) in the mouse lung tissues treated with silica. Red represents liposomes; Green represents α-SMA or E-cadherin.

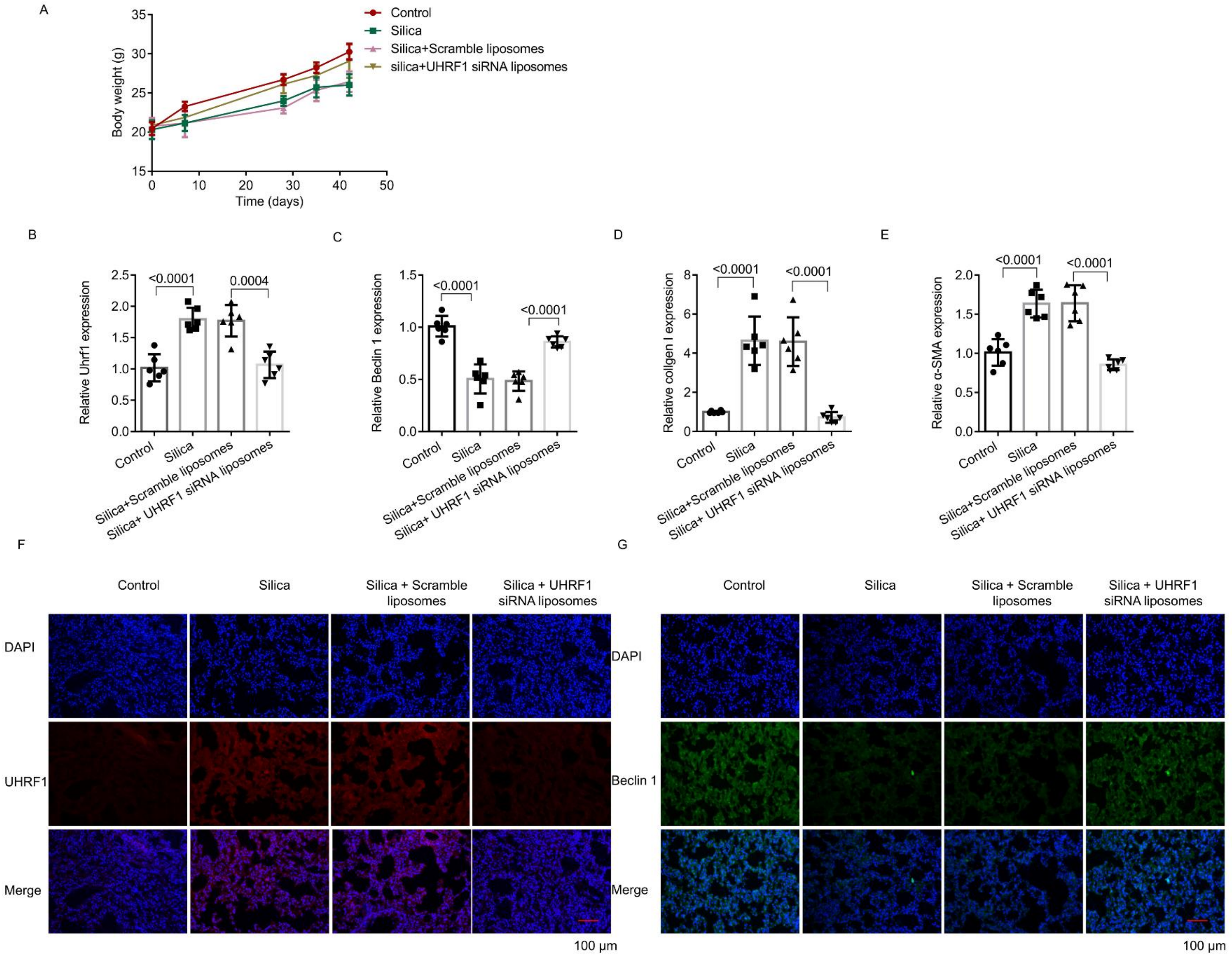

Fig. S8. Administration of UHRF1 siRNA liposomes attenuates silica-induced pulmonary fibrosis in mice.

(A) The body weight of the mice in each group, n = 6 in each group. (B-E) qRT-PCR detection of UHRF1, Beclin 1, α-SMA and Collagen I expression in lung tissues in the different groups. Data are shown as mean ± SEM (n = 6 in each group). (F-G) Immunohistochemical staining of UHRF1 and Beclin 1 in mouse lung tissues for the indicated groups. UHRF1 stained red; Beclin 1 stained green; DAPI stained blue. (B-E) *P* values were from a one-way ANOVA post hoc test with Tukey

correction.

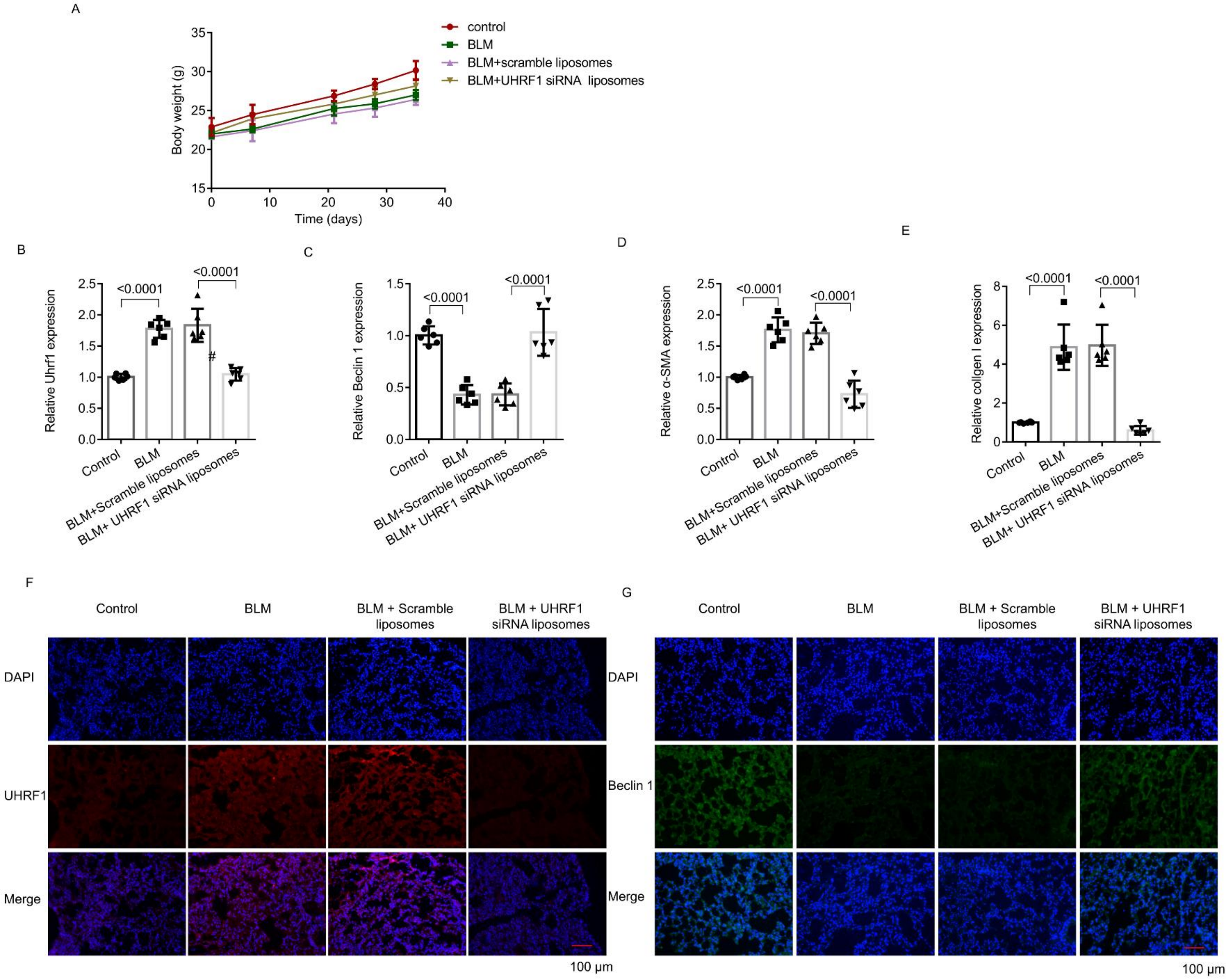

Fig. S9. Administration of UHRF1 siRNA liposomes attenuates BLM-induced pulmonary fibrosis in mice.

(A) The body weight of the mice in each group, n = 6 in each group. (B-E) The expression of UHRF1, Beclin 1, α-SMA and Collagen I expression in lung tissues was detected by qRT- PCR assay. Data are shown as mean ± SEM (n = 6 in each group). (F-G) Immunohistochemical staining of UHRF1and Beclin 1 in mouse lung tissues for the indicated groups. UHRF1 stained red; Beclin 1 stained green; DAPI

stained blue. (B-E) *P* values were from a one-way ANOVA post hoc test with Tukey correction.

Supplementary table1

Genn Name	Forward primer (5'-3')	Reverse primer (3'-5')
UHRF1 (Homo)	AACTGCTTTGCTCCCATCA	TCTTGCCACCCTTGACATT
Beclin 1 (Homo)	GGATGGTGTCTCTCGCA	CAGTCTTCGGCTGAGGTT
TEAD1 (Homo)	AGAAAGTTGAGGCCAGAGG	GGACCAAAGTGGCAGGA
TEAD4 (Homo)	CCACAAGCTCAAGCACCTC	TCACTGGCTGACACCTCAA
YAP (Homo)	CCGTTTCCCAGACTACCTT	TTGGCATCAGCTCCTCTC
GAPDH (Homo)	CCTTCCGTGTCCCCACT	GCCTGCTTCACCACCTTC
Uhrf1 (Mus)	CCAAGAATGTCCGTGCTC	GGTTTGGCGCTTCCTAC
Beclin 1 (Mus)	CTGTAGCCAGCCTCTGAAA	CCTCTTCCTCCTGGGTCT
Tead1 (Mus)	GACACACGCACCTCTTTG	GGATGAGGGATGCTGCT
Tead4 (Mus)	TGCAGAAAGAACAAAGCCA	CATCGGTGCCTGAGAATG
Yap (Mus)	GCTTCCCCGACTACCTG	CACAGACTCCACGTCCAA
Gapdh (Mus)	TGTTTCCTCGTCCCGTAGA	ATCTCCACTTTGCCACTGC

Primer Sequence for RT-qPCR

Gene Name	sense	antisense
UHRF1	AGACGGAAUUGGGGCUGUATT	UACAGCCCCAAUUCCGUCUTT
Beclin 1	CUGGACACGAGUUUCAAGATT	UCUUGAAACUCGUGUCCAGTT

Sequences of UHRF1 siRNA and Beclin 1 siRNA

Supplementary table2

Primers	Sequence (5'-3')
UHRF1 -CHIP-F	CGCGGAACAGTCTTGTGA
UHRF1 -CHIP-R	GGCCCCTTCTTGGTCATT
Beclin 1 -CHIP-F	CTCAGGAGAGGAGCCATTT
Beclin 1 -CHIP-R	CTGCGAGAGACACCATCC

Primer Sequence for ChIP-qPCR

Supplementary table3

Primers	Sequence (5'-3')
Beclin 1(M)	F: TTTGGAGTAGTTGGGATTATAGGC
	R: AAAAACCAAACAAAATTACTCACG
Beclin 1(U)	F: TTTGGAGTAGTTGGGATTATAGGTG
	R: AAAAACCAAACAAAATTACTCACAC

Primer Sequence for MSP

M: methylation; U: unmethylation
